# Transforming the Buckyball: Regioselective Synthesis of Water-Soluble [60]Fullerene Derivatives for Biomedical Applications

**DOI:** 10.3390/molecules31122005

**Published:** 2026-06-08

**Authors:** Olga A. Kraevaya, Pavel A. Troshin

**Affiliations:** 1 Federal Research Center for Problems of Chemical Physics and Medicinal Chemistry of the Russian Academy of Sciences, Semenov Prospect 1, Chernogolovka 142432, Russia; okraevaya@inbox.ru; 2 Zhengzhou Advanced Research Institute of Harbin Institute of Technology, No. 112 Longhu Zhonghuan North Road, Jinshui District, Zhengzhou 450000, China

**Keywords:** water-soluble fullerene derivatives, biological activity, biomedical applications, synthesis, isomerically pure fullerenes

## Abstract

Water-soluble fullerene derivatives exhibit a wide range of fascinating biological properties, including antioxidant, antiviral, antitumor, antibacterial, and myogenic effects. During the initial stage of research, most of the reported data on the biological activity of fullerenes were obtained using complex, inseparable mixtures of regiomers with a big focus on fullerenols as the most accessible form of water-soluble fullerene-based compounds. However, during the past decade, significant progress has been made in the synthesis of various isomerically pure water-soluble fullerene derivatives, which opens up possibilities for more directed investigations of their biological activity. In this review, we will highlight current methods for the straightforward synthesis of different types of water-soluble fullerene derivatives with well-defined molecular structures. Special attention will be paid to the possibilities of the precise control of the number, types, and positions of functional groups on the fullerene cage. We will also discuss the opportunities for and challenges within the biomedical applications of water-soluble fullerene derivatives.

## 1. Introduction

Fullerenes are well known as the first isolated stable molecular form of carbon, which not only launched a new field of organic chemistry but also, through the development of carbon nanotubes and other carbon forms, a new field of materials science [1].

Soon after their discovery during mass spectrometry analysis of the products of graphite laser evaporation, fullerenes C_60_ and C_70_ became available at the gram scale using the approach proposed by Huffman and Krätschmer in 1990 [2].

**Figure 1:**
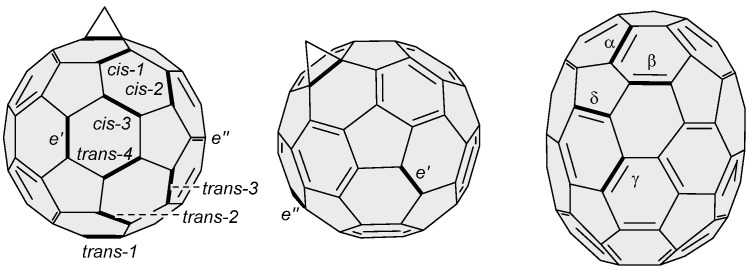
Positional isomerism of the fullerene C_60_ bis-adducts and types of double bonds in C_70_.

After the problem of fullerene mass production and purification was solved, the era of covalent modification of the fullerene cage began, and other important fundamental challenges have emerged. Namely, it was discovered that the uncontrolled addition of functional groups to the fullerene cage often led to the formation of complex mixtures of regioisomers and derivatives with different numbers of addends. For example, monofunctionalized C_60_ has nine symmetrically non-equal 6-6 bonds that can react in a second addition (Figure 1) [3]. *E,e,e*-isomers are the most prominent members in the family of tris-substituted fullerenes, since they can be rather selectively synthesized under specific reaction conditions [4,5]. The functionalization of C_70_ is even more challenging. Unlike C_60_—whose perfect spherical geometry yields just one possible mono-adduct isomer—C_70_ with *D_5h_* symmetry has four inequivalent types of [6,6]-bonds defined as α, β, γ, and δ, leading to the C_70_-based bis-adducts having 38 possible isomers on the condition of identical groups and additions occurring exclusively on [6,6]-bonds [6,7]. This inherent complexity means that the regioselective bis-functionalization of C_70_ is a considerably more difficult task than it is for C_60_.

This limitation led most researchers to focus on synthesizing monofunctionalized fullerene derivatives—products of addition reactions involving only one double bond of C_60_. Since all double bonds on the fullerene cage are equivalent, these derivatives can be readily isolated as pure compounds. Classical functionalization methods, such as the Bingel–Hirsch [4] and Prato [8] reactions, were developed and widely adopted for this purpose. Some of the pioneers of fullerene chemistry were willing to embrace the complexity of multiple functionalization of the fullerene cage, which led to the discovery of novel reactions and addition patterns in fullerene chemistry.

Prof. R. Taylor and his team from the University of Sussex have left a remarkable legacy on the arylation [9,10], alkylation [11], alkoxylation [12], halogenation [13,14] of fullerenes and further transformation of halogenated fullerenes. Prof. L. Gang from Peking University has contributed to the peroxide chemistry of fullerenes [15] and has developed unique methods for the synthesis of open-cage [16,17], endohedral [18] and heterofullerenes [19]. Prof. E. Nakamura and colleagues discovered the unique pentaaddition reaction of organocopper reagents to C_60_ [20,21]. Prof. S. Troyanov has developed an efficient high-temperature ampule synthesis method for a broad variety of highly functionalized derivatives of fullerenes and endometallofullerenes and succeeded in the X-ray structural characterization of >100 unique compounds bearing different halogen atoms and/or perfluroalkyl groups attached to the carbon cage [22,23,24,25].

The extreme hydrophobicity of pristine fullerenes prevents their effective dispersion in physiological fluids, hindering in vivo applications. The addition of hydrophilic polar groups, which are capable of hydrogen bonding with water molecules, is the key to achieving water solubility. This functionalization not only confers water solubility but also enhances bloodstream retention time by reducing immune system recognition of the fullerene derivatives as foreign particles [26].

The number, chemical nature, and relative position of organic addends attached to the fullerene cage determine solubility in aqueous media, tendency to aggregation, biological activity profile, and the electron affinity of fullerene derivatives.

To obtain truly water-soluble covalently modified fullerene derivatives, several polar hydrophilic groups have to be attached to the cage directly or through a linker. Depending on the type of the solubilizing groups, fullerene derivatives can be divided into cationic (mostly ammonium salts) or anionic (with attached carboxylic, phosphonate, sulfonate or hydroxyl groups). Typically, good solubility in water can be reached when the number of ionic groups exceeds four. For example, potassium salts of fullerene-based polycarboxylic acids and aminofullerenes with five cationic groups were reported to have high solubility in water (>50 mg/mL) [27]. Non-ionic individual fullerene derivatives, such as, for example, fullerene-based polyols with 24 hydroxyl groups reach solubilities of up to 75 mg/mL [28].

**Figure 2:**
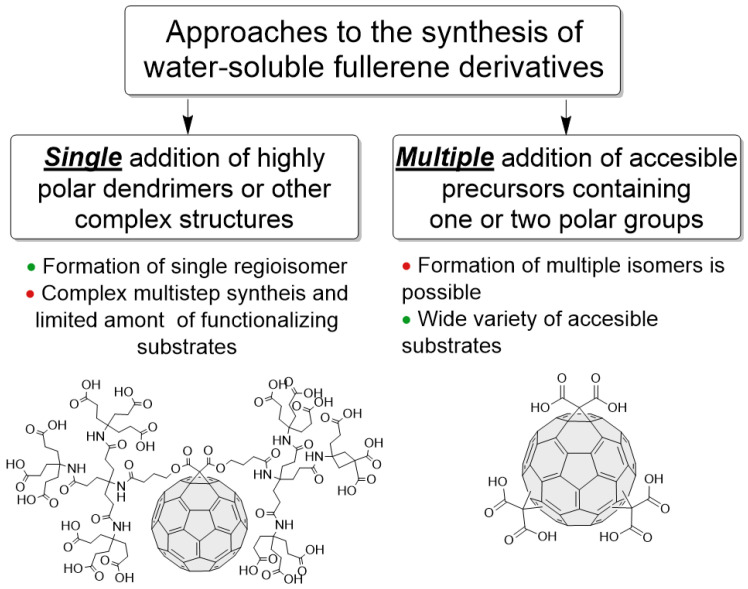
Two different approaches to the synthesis of the water-soluble fullerene derivatives.

Thus, two different approaches to the synthesis of individual water-soluble fullerene derivatives can be proposed: (1) to utilize a branched reagent that has a required amount of hydrophilic groups in its structure and can be attached via functionalization of only one fullerene double bond; (2) to use a substituent bearing one or two polar functionalities and a protocol that provides a regioselective addition of several addends (Figure 2).

The first approach was utilized to obtain one of the first water-soluble individual dendro [60]fullerene derivatives containing 18 carboxyl groups using the reaction of C_60_ with branched malonate [29,30]. This compound was reported as one of the most effective inhibitors of HIV-1 protease in vitro.

However, the limitations of this method, based mostly on the low accessibility of dendrite hydrophilic precursors, were soon realized, and focus was shifted to the second approach.

Thus, the search for regioselective methods for the multiple functionalization of the fullerene cage is still underway and represents an important synthetic task.

In this review, we will introduce general approaches to the synthesis of individual fullerene derivatives (Section 2), discuss the most popular synthetic routes to water-soluble fullerene-based compounds, such as Bingel–Hirsch (Section 3) and Prato (Section 4) reactions, as well as the addition of organocopper reagents (Section 5). In addition, we will discuss less conventional approaches based on the substitution of halogen atoms in halogenated fullerenes (Section 6). We will also consider protocols that allow one to obtain water-soluble fullerenes with different types of functional addends, tailoring à la carte the properties of the resulting molecules (Section 7).

Finally, we will showcase the most promising results from investigations of the biological activity of individual fullerene derivatives (Section 8).

## 2. General Approaches to the Synthesis of Isomerically Pure Fullerene Derivatives

Despite the fact that the synthesis of numerous individual fullerene derivatives has been reported, most of them are devoted to the preparation of mono-adducts, while the simple and selective synthesis of polyaddition products still remains a non-trivial task.

Classical approaches to the functionalization of the fullerene cage allow the synthesis of compounds with 1–2 addends with acceptable selectivity. Attempts to use these methods for the synthesis of polyfunctionalized fullerene derivatives often lead to the formation of inseparable mixtures of isomers [31].

**Figure 3:**
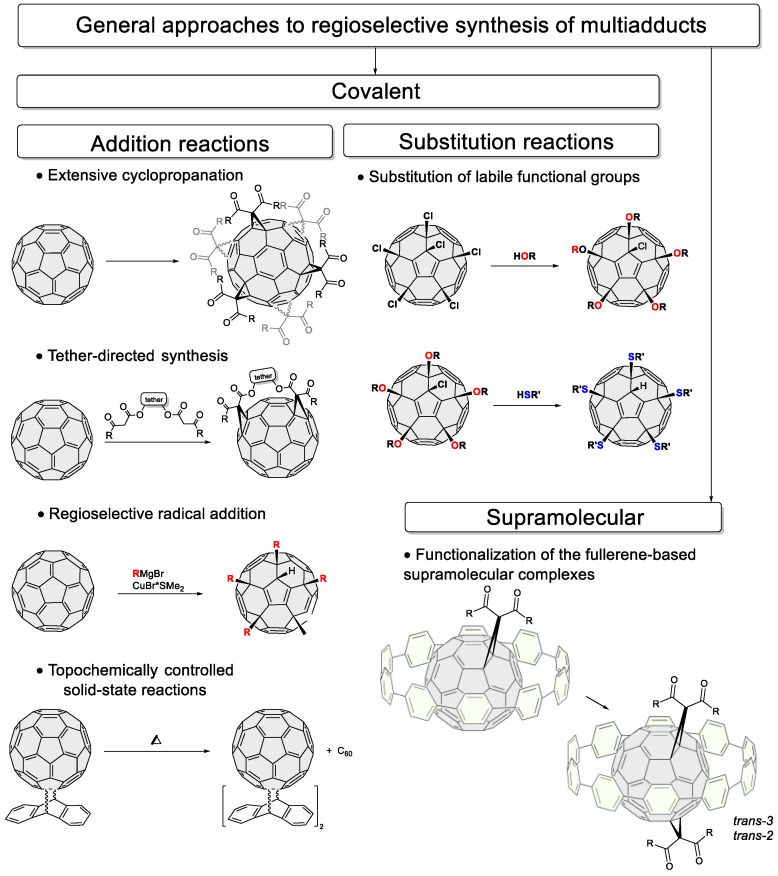
General approaches to regioselective synthesis of fullerene polyadducts.

The problem of the regioselective attachment of multiple addends to the fullerene cage is being actively addressed using various approaches, which can be formally divided into covalent and supramolecular (Figure 3).

The covalent approach includes methods based on both addition and substitution reactions. Several examples of regioselective addition reactions are presented in Figure 3. For example, extensive cyclopropanation of the fullerene cage, which involves five or six double bonds, leads to the selective formation of a single isomer in each case. Covalent methods that use different “guiding” spacers to provide the regioselective formation of bis- and tris-adducts of fullerenes have been actively elaborated [31].

Methods based on removable templates, such as anthracene attached to the fullerene cage, have been proposed. Template molecules are often removed using a retro-Diels–Alder reaction. This approach can be illustrated by the conversion of the bis-anthracene adduct of fullerene into *e,e,e,e*-C_60_[C(CO_2_Et)_2_]_4_ [32,33].

Another regioselective method of covalent functionalization is based on the fusion of C_60_ [34] and C_70_ [35] fullerenes with anthracene at high temperature, during which so-called topochemical orthogonal transposition occurs. The lattice of a fullerene derivative can serve as a template that fixes reagent molecules in the necessary position. Thus, heating a crystalline adduct of [60]fullerene with anthracene afforded *trans*-1-bis-adduct and fullerene in virtually quantitative yields. Such an unexpected mechanism was associated with the packing of molecules in the crystal according to the ‘head-to-tail’ type.

The regioselective synthesis of fullerene derivatives via substitution reactions represents a very convenient and versatile approach but also has several limitations, which will be discussed later in this article. Since the regioselective chlorination of fullerenes with the formation of the products C_60_Cl_6_, C_70_Cl_8_ and C_70_Cl_10_ has been reported, these molecules became popular precursors for the synthesis of polyfunctional fullerene derivatives through the replacement of labile chlorine atoms with organic addends bearing hydrophilic groups [36]. Alkoxy substituents were also reported as good leaving groups that can be substituted by thiol residues on the fullerene cage [37].

The supramolecular approach is based on the preliminary preparation of host–guest fullerene complexes with cycloparaphenylenes and other macrocyclic molecules, followed by the regioselective functionalization of the resulting complexes [33]. The limitations of these approaches include the specific chemical nature of substrates, multi-stage processes, and the use of complex structures to obtain supramolecular complexes.

The supramolecular approach was actively developed by several research groups, including the teams of Prof. X. Ribas and Prof. M. Costas (Universitat de Girona) [38], Prof. Y. Xu (Henan University) and Prof. M von Delius (Ulm University) [39], and Prof. G.H. Clever (TU Dortmund University) [40].

For example, the complex between C_60_ or its derivatives and [10]CPP represents the perfect fit regarding the size of guest and host (Figure 3) [41]. The addition of one equivalent of [10]CPP to fullerene monomalonate leads to the corresponding inclusion complex, which yields two major isomers (*trans*-2 and *trans*-3) upon addition of the next malonate equivalent [42].

Another example of a supramolecular approach is the complexation of a supramolecular tetragonal prismatic nanocapsule bearing two Zn-porphyrin units and four macrocyclic Pd-based molecular clips with C_60_, C_70_, and C_84_ [43]. This nanocapsule was later used as a supramolecular mask, leading to the formation of tetrakis-adduct (*D*_2*h*_-symmetric *e,e,e,e*-regioisomer).

Another fascinating example of the supramolecular approach has been recently reported, which allowed for the selective preparation of fullerene hetero-tris-adducts. After the first addend was introduced onto C_60_ through a cascade process involving Ru-catalyzed cycloisomerization of 1,6-enynes followed by a Diels–Alder reaction, the supramolecular mask was introduced and two pentacene addends were regioselectively attached [44].

The combination of supramolecular and covalent chemistry of fullerene yielded a series of beautiful supramolecular fullerene-based architectures, including [10]cycloparaphenylene-fullerene [2]rotaxanes [42], matryoshka-like complexes, etc. [45]. One of the pioneers in this field, Prof. F. Diederich, systematically explored the chemistry of fullerenes with a focus on functional and switchable supramolecular nanosystems [46]. The history of the development of this research area and its perspectives are summarized in several review papers [39]. In this review article, we only cover the basic idea of the supramolecular approach, since methods utilized for the further functionalization of supramolecular fullerene complexes are similar to those discussed below.

## 3. Cyclopropanation Reactions for the Synthesis of Individual Water-Soluble Fullerene Derivatives

**Figure 4:**
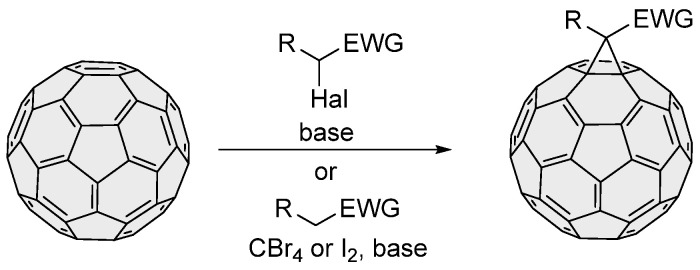
Bingel–Hirsch cyclopropanation reaction.

The protocol for the cyclopropanation of the fullerene developed by C. Bingel and A. Hirsch is one of the most popular approaches for functionalized derivatives (Figure 4) [4]. This reaction is widely utilized for the synthesis of water-soluble fullerene derivatives, including mono- and poly-adducts. From the perspective of medicinal chemistry, monoadducts and hexakis-adducts appear to be particularly relevant, due to their occurrence as single regioisomers.

**Figure 5:**
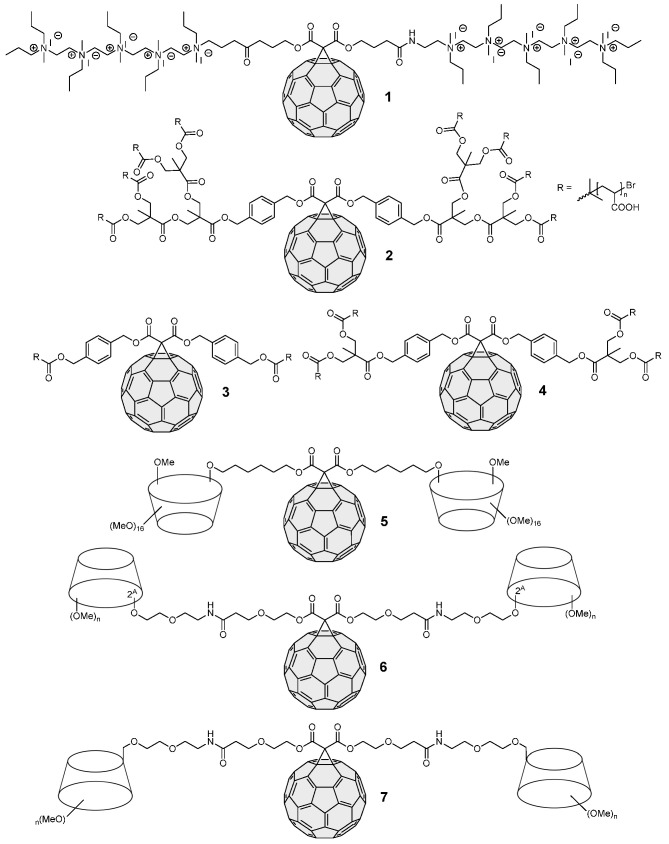
Water-soluble fullerene mono-adducts obtained using Bingel–Hirsch cyclopropanation.

Polycationic, polyanionic, and neutral mono-adducts have been obtained over the years (Figure 5). Namely, water-soluble decacationically armed C_60_ decaiodide mono-adduct **1** was obtained via the Bingel–Hirsch approach. The authors also reported that it can be applied as a photosensitizer and potentially a nano-PDT (photodynamic therapy) agent against pathogenic bacteria and cancer cells [47]. Highly water-soluble C_60_-anchored multiarmed poly(acrylic acids) **2**–**4** were obtained through the complex multistep synthetic protocol involving fullerene cyclopropanation with a polymeric malonate precursor [48]. Another approach to the water-soluble fullerene was based on the synthesis of fullerene–cyclodextrin conjugates (compounds **5**–**7**) [49,50].

Further cyclopropanation of mono-adducts with the formation of 2 to 4 cyclopropane rings on the fullerene cage yields mixtures of isomeric products that require chromatographic separation (Figure 1). For bis-adducts, the formation of nine isomers is possible if addends are different, and eight isomers can be obtained if two identical addends are attached. Cyclopropanation of *trans*-3-bis-adduct (*trans-3*-C_60_A_2_) results in the preferential formation of a *D*_3_-symmetrical tris-adduct *trans*-3-C_60_A_3_, in which all three addends are in *trans*-3-positions.

**Figure 6:**
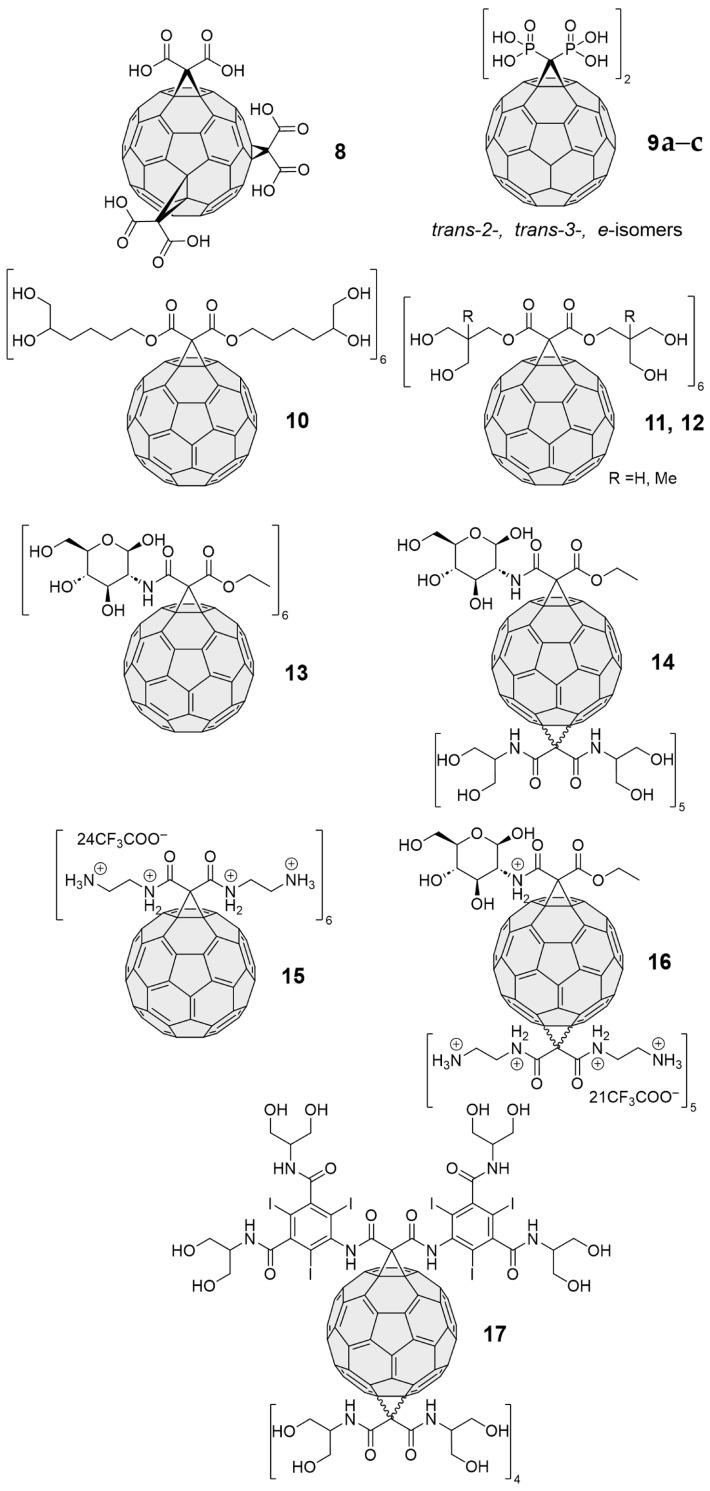
Examples of individual fullerene multiadducts obtained via cyclopropanation reaction.

The first actual synthesis of water-soluble C_60_ can also be attributed to Lamparth and Hirsch [51], who emphasized the critical importance of isolating single regioisomers for biomedical evaluation. They synthesized polycarboxylic tris-adducts *C*_3_-C_60_A_3_ (compound **8**) and *D*_3_-C_60_A_3_ via NaH-promoted hydrolysis of the respective malonic esters. Respective malonic ethyl esters were obtained using cyclopropanation of individual bis-adducts followed by HPLC-assisted purification [4]. These tris-adducts have been extensively studied for a variety of biomedical applications (Figure 6) [52,53].

Water-soluble compounds **9a**–**c** functionalized with two diphosphonate groups were obtained via hydrolysis of the respective chromatographically separated isomeric ethyl esters [54].

Fortunately, for polyadducts with five and six cyclopropane rings, only one isomer is possible. This tendency was actively used for the synthesis of individual water-soluble penta- and hexa-adducts of fullerenes, such as compounds **10**–**16**. Polyol methanofullerenes **10**–**12** were obtained via exhaustive cyclopropanation of C_60_ with acetonide-protected precursors, followed by deprotection under acidic conditions [28].

The water-soluble glycofullerenes **13** and **14** containing D-glucosamine fragments were synthesized using the two-step modified Bingel–Hirsch methodology [55].

Polycationic fullerene hexakis adducts **15** and **16** were also obtained using the approach based on exhaustive or two-step cyclopropanation of the fullerene cage [56].

To obtain the first fullerene-based X-ray contrast agent **17**, a single addend containing six iodine atoms and eight protected hydroxyl groups was appended to C_60_ followed by the addition of four more addends, each containing four protected hydroxyl groups. Final deprotection afforded the perfectly water-soluble (>460 mg mL^−1^), non-ionic, highly iodinated (24% of iodine) individual fullerene derivative [57].

**Figure 7:**
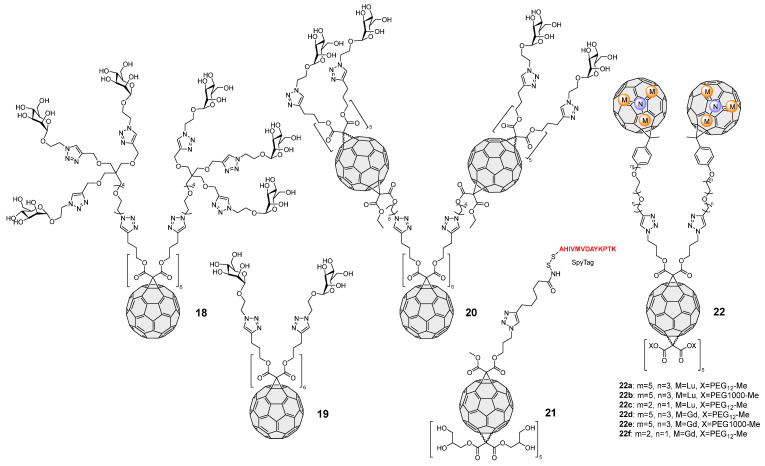
Examples of individual fullerene multiadducts obtained via CuAAC conjugation.

The development of click chemistry gave a new direction to the synthesis of water-soluble fullerenes. The copper-catalyzed azide–alkyne [2+3] cycloaddition (CuAAC) approach was utilized for the synthesis of fullerene conjugates with carbohydrates, such as compounds **18** [58], **19** [59] and **20** (Figure 7) [60], bearing mannose residues.

Since the cyclopropanation reaction allows for the regioselective synthesis of C_60_A_5_A′ hexakis adducts with two different types of addends, this synthetic method was used for the CuAAC conjugation of functionalized water-soluble fullerene C_60_A_5_ moiety with biological substrates such as peptides (compound **21**) and other fullerene-based residues (compound **22**), thus producing supramolecular structures and so-called giant superballs [61,62].

J. Zhang and colleagues reported the synthesis of metallobuckytrios, three buckyball systems, obtained via a CuAAC click reaction (compounds **22a**–**f**) (Figure 7) [63].

## 4. [2+3]Cycloaddition Reactions for the Synthesis of Individual Water-Soluble Fullerene Derivatives

**Figure 8:**
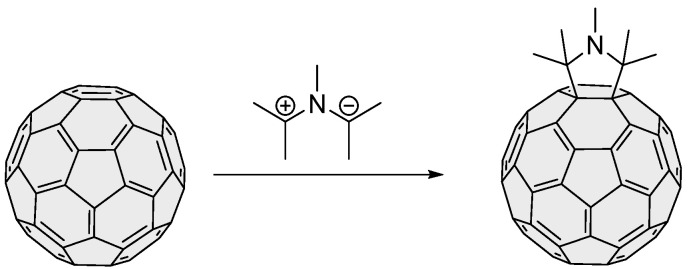
Synthesis of fulleropyrrolidines via addition of azomethine ylides.

Fullerene C_60_ readily undergoes [2+3] cycloaddition with different 1,3-dipolar reagents, namely, azomethine ylides, azides, nitrile ylides, diazo compounds, nitrile imines, thiocarbonyl ylides, nitrile oxides, and carbonyl ylides [64]. Among those reactions, the method developed by Prof. M. Prato and based on the addition of azomethine ylides to the fullerene cage is one of the most convenient and high-yielding (Figure 8) [8]. It affords fulleropyrrolidines and is often utilized for the synthesis of water-soluble fullerenes. Azomethine ylides are typically obtained from α-amino acids or their derivatives and carbonyl compounds [65]. Similarly to other mono-adducts, [2+3]cycloaddition products with a single pyrrolidine unit annealed to the [60]fullerene cage produce only one regioisomer. In a comparative analysis of multiaddition regioselectivity, Lu et al. demonstrated that bis-addition via the Prato reaction offers less control over product formation compared to the Bingel–Hirsch reaction [66]. Additional difficulty in individual product purification arises from the *cis/trans* isomerism present in pyrollidinofullerenes due to the chiral carbon atoms in the ring. This becomes particularly important for biomedical applications, as even slight differences in molecular structure (stereoisomerism) can dramatically alter how these fullerene compounds behave in living systems, affecting pharmacokinetics and pharmacodynamics [67].

An early attempt to synthesize water-soluble fullerenes via the Prato reaction was not completely successful [68]. The introduction of two triethylene glycol chains on the pyrrolidine ring of the mono-adduct (compound **23**) gave a poor solubility of 3 × 10^−5^ M in a 9:1 water-DMSO mixture [69].

**Figure 9:**
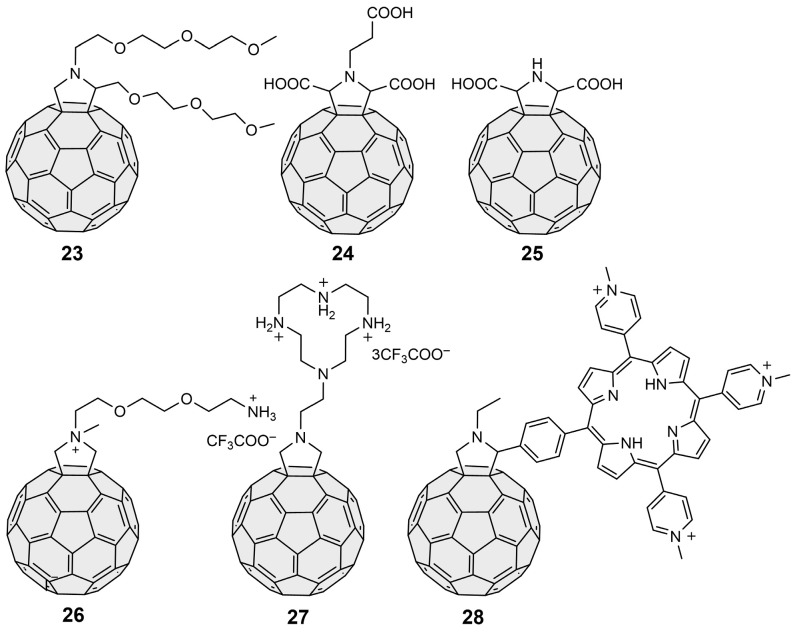
Several examples of water-soluble monofunctionalized fulleropyrrolidines.

However, the introduction of several ionic groups such as carboxylic and positively charged amines (compounds **24**–**28**) provided higher solubility of the monofunctionalized fulleropyrrolidines in water (Figure 9).

A recent review by Zhang et al. summarizes the data on the biological application of fullerene mono-adducts [70].

**Figure 10:**
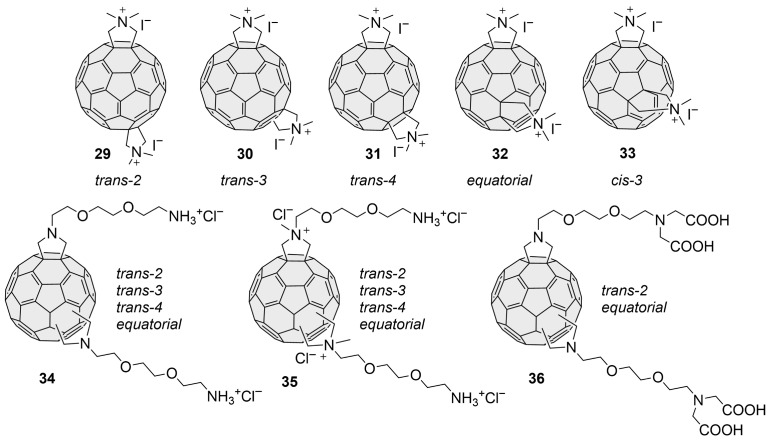
Examples of individual fullerene multiadducts obtained via [2+3]cycloaddition reaction.

Isomerically pure fulleropyrrolidines with multiple addends represent a versatile class of materials with tailored properties for a wide range of applications. Marchesan et al. synthesized isomeric bis-fulleropyrrolidinium salts (**29**–**36**) and pegylated polycationic fullerene derivatives (Figure 10) [71,72].

## 5. Selective Multiaddition of Organocopper Reagents to Fullerene

**Figure 11:**
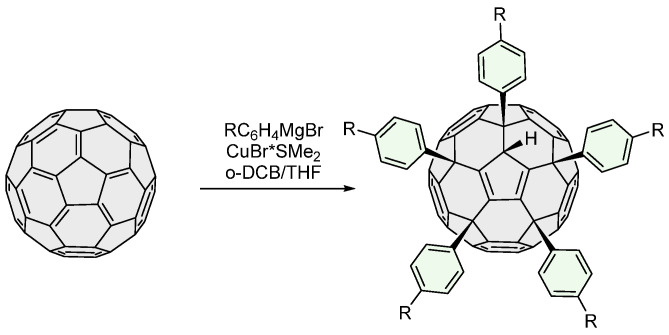
Synthesis of penta(aryl)[60]fullerenes via the addition of phenylcopper reagents.

In 1996, Prof. E. Nakamura and colleagues reported that a phenylcopper reagent derived from a Grignard reagent and a copper(I) halide adds five times to [60]fullerene and with a complete regioselectivity in quantitative yield to produce penta(aryl)[60]fullerenes C_60_Ar_5_H (Figure 11) [20,73]. Synthesized pentaaryl [60]fullerene anions C_60_Ar_5_^−^·K^+^ were reported to be soluble in water and form spherical bilayer vesicles [74].

**Figure 12:**
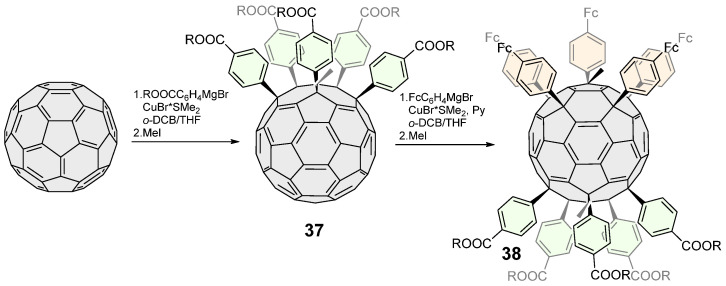
Synthesis of deca(aryl)[60]fullerenes.

Pyridine-modified organocopper reagents are used for single-step decaaddition reactions to [60]fullerene to obtain deca(organo)[60]fullerenes C_60_R_10_H_2_ (Figure 12) [73]. Importantly, this approach also allows for the sequential addition of two different types of aromatic addends. The developed protocol was efficiently utilized for the synthesis of deca(aryl)[60]fullerenes bearing five ferrocenyl and five ester groups [74]. Thus, it can also be used for the synthesis of water-soluble fullerene derivatives with unique addition patterns.

**Figure 13:**
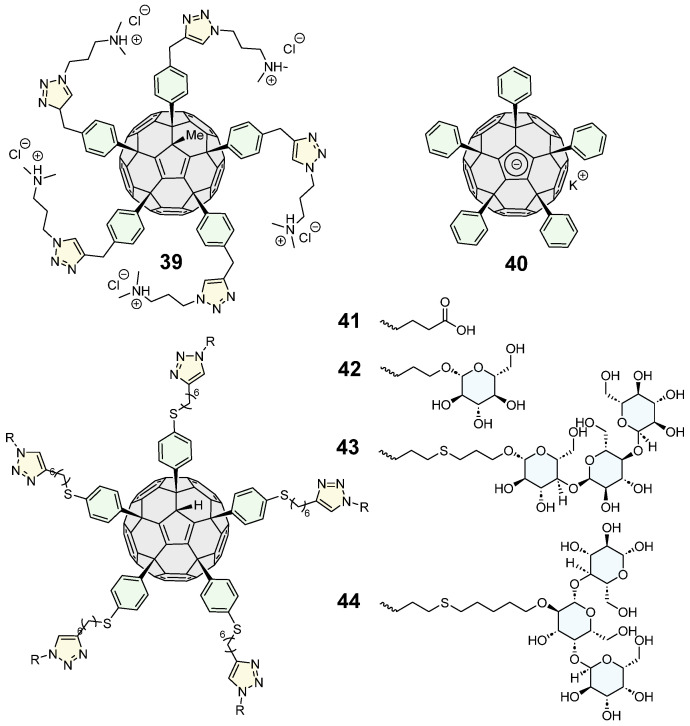
Fullerene derivatives synthesized via selective multiaddition of organocopper reagents.

Importantly, the regioselective multiaddition of organocopper reagents to the fullerene cage allowed for the introduction of useful functional groups such as hydroxyl, thiol, ester, and carboxylic groups. Examples of neutral, anionic, and cationic fullerene derivatives, obtained via the addition of organocopper compounds, are presented in Figure 13 [75,76,77].

## 6. Chlorinated and Fluorinated Fullerenes as Precursors for the Synthesis of Water-Soluble Fullerene Derivatives

### 6.1. Chlorofullerenes as Precursors for Water-Soluble Fullerene Derivatives

Chlorofullerenes C_60_Cl_n_ were obtained in 1991, thus becoming one of the first isolated fullerene derivatives [78,79]. In 1993, the group of Prof. R. Taylor synthesized and characterized isomerically pure chlorofullerene C_60_Cl_6_ using the reaction of fullerene with iodine monochloride in benzene [13]. Since then, the method for the synthesis of C_60_Cl_6_ has been optimized, chlorination reaction intermediates have been isolated and characterized, and the mechanism of the chlorination reaction has been proposed [80].

**Figure 14:**
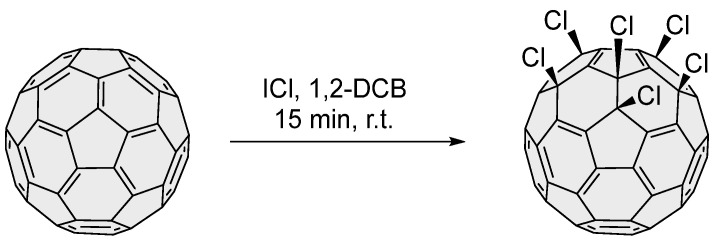
Facile synthesis of chlorofullerene C_60_Cl_6_.

The optimized procedure (Figure 14) allows the quick synthesis of pure C_60_Cl_6_ (>97%, HPLC) with quantitative yields starting from the solution of C_60_ in 1,2-dichlorobenzene [81]. The resulting compound is soluble in aromatic solvents such as toluene and chlorobenzenes.

A similar procedure utilizing C_70_ as a precursor allows us to obtain C_70_Cl_8_, which has the distinct feature of being insoluble in most organic solvents. However, it is possible to overcome this limitation using either a soluble analog C_70_Cl_10_ or performing the heterogeneous reaction under elevated temperatures with the gradual substitution of Cl atoms and solubilization of the formed products [14].

Various halogenofullerenes have been considered as precursors for the synthesis of functionalized fullerenes via substitution reactions. However, isomerically pure fluorinated fullerenes are not as easily accessible, bromofullerenes have limited stability and are poorly soluble, and iodofullerenes have not been isolated. Thus, chlorofullerene C_60_Cl_6_ is the optimal precursor for the synthesis of functional fullerene derivatives because it simultaneously possesses the following required properties: (1) can be quickly and easily obtained in isomerically pure forms with high yields in gram quantities; (2) has high solubility in organic solvents; (3) chlorine atoms in the structure can be easily substituted by various organic addends; (4) the original C_s_-symmetrical (cyclopentadienyl-type) halogen addition pattern is preserved during the substitution reactions, allowing the regioselective formation of functional derivatives; (5) six introduced addends is enough to easily change the properties of the molecule (including solubility in water) in a desired manner via substitution reactions.

Additionally, C_60_Cl_6_ has a specific reactivity due to the presence of two chlorine atoms in the 1,2-position, allowing the selective replacement of five chlorine atoms with bulky addends, while the sixth halogen atom is typically not substituted due to steric hindrance. The substitution of the remaining chlorine atom can be carried out during further synthetic steps, which opens up possibilities for the synthesis of compounds with several types of addends. This approach will be discussed further in detail.

The chemical properties of C_60_Cl_6_ have been investigated rather intensively during previous years. Most of the approaches that allow us to obtain water-soluble fullerene derivatives from C_60_Cl_6_ are based on its reactions with nucleophiles containing hydrophilic groups in their structures.

### 6.2. Reactions with Amines and Amino Acids

**Figure 15:**
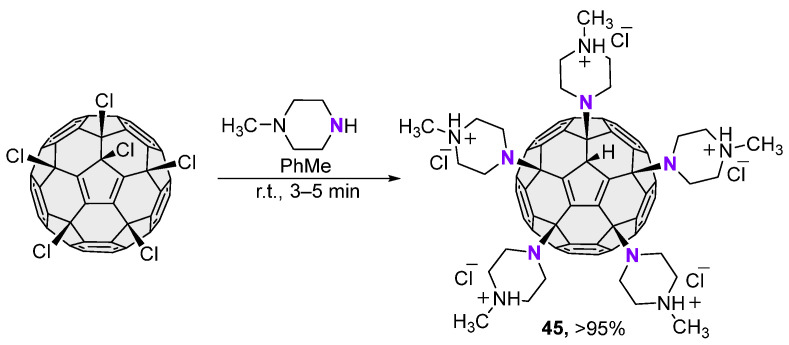
Preparation of polycationic water-soluble fullerene derivative with attached N-methylpiperazine hydrochloride residues.

The reaction of C_60_Cl_6_ with amines can be utilized as a method for the synthesis of water-soluble fullerene derivatives if ammonium groups are formed during the synthesis or during further quaternization or protonation reactions. For example, the interaction of C_60_Cl_6_ with N-substituted piperazines can be used as an effective method for the synthesis of polycationic water-soluble fullerene derivatives (Figure 15). The reaction is performed in toluene and leads to the immediate precipitation of polycationic products, which have shown exceptionally high solubility in water (>300 mg mL^−1^) [82].

Amino acid-functionalized water-soluble fullerene derivatives represent a particularly promising class of compounds, combining the inherent biological compatibility of amino acids with the diverse bioactive potential of fullerene systems.

**Figure 16:**
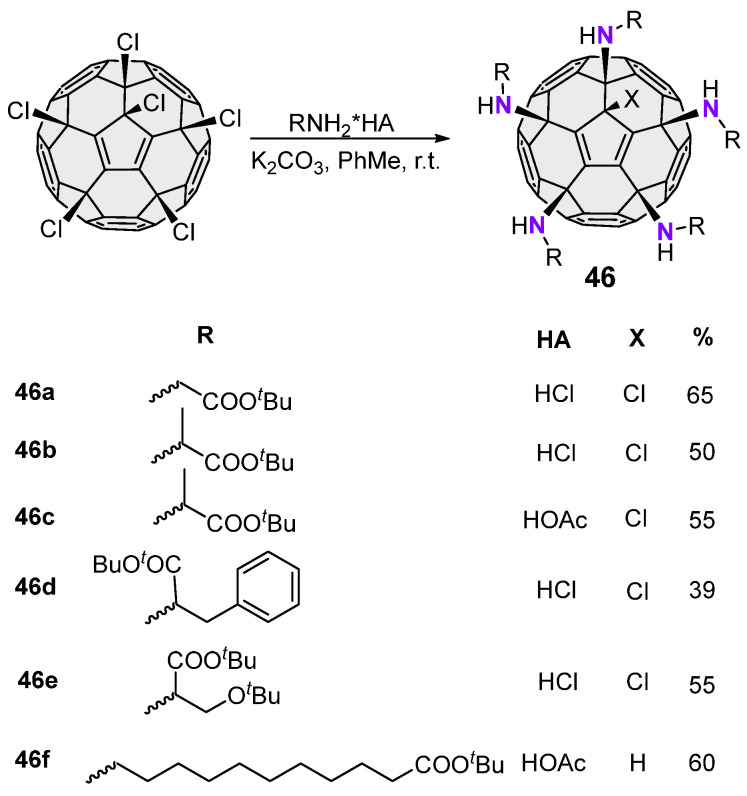
Reaction of C_60_Cl_6_ with hydrochlorides and hydroacetates of tert-butyl esters of amino acids.

While initial synthetic efforts primarily produced complex mixtures of fullerene–amino acid adducts [83,84,85], recent advances have enabled the preparation of isomerically pure derivatives. They can be obtained via the reaction of C_60_Cl_6_ with tert-butyl esters of amino acids followed by the cleavage of tert-butyl groups with a strong acid such as trifluoroacetic (Figure 16) [86].

Chlorofullerene C_60_Cl_6_ also reacts with methyl esters of amino acids, forming compounds with similar structures. These functionalized fullerenes have been successfully employed to construct drug-loaded vesicles, demonstrating effective encapsulation of clinically relevant anticancer agents, including 5-fluorouracil, cyclophosphamide, and cisplatin [87]. However, methyl esters are not suitable for the synthesis of highly soluble polycarboxylic derivatives due to the absence of a synthetic protocol allowing the deprotection of ester groups without degrading the C-N bonds between the fullerene cage and amino acid-based addend.

Although reactions of chlorofullerene with N-nucleophiles allow us to obtain a wide variety of water-soluble derivatives, the formation of C-N bonds between the carbon cage and solubilizing addends is undesirable due to the comparatively low stability of these compounds in aqueous media. Aqueous solutions of fullerene derivatives with C-N bonds between the cage and addend typically degrade through partial hydrolysis and the depletion of attached residues, which leads to a loss of solubility. Thus, the development of alternative approaches to the synthesis of amino acid- and protein-modified fullerene derivatives is of great importance.

### 6.3. Reactions with Alcohols and Hydroxy Acids

**Figure 17:**
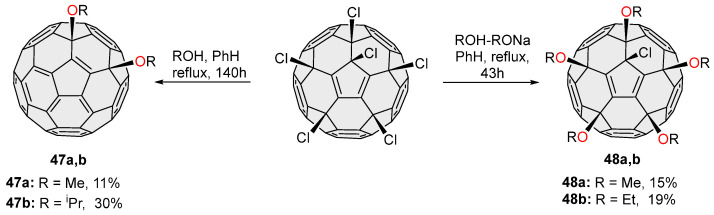
Reactions of C_60_Cl_6_ with alcohols and alkali metal alcoholates.

The reaction of chlorofullerene with alcohols was one of the first reactions discovered and reported for chlorofullerenes. Prof. R. Taylor and colleagues demonstrated that prolonged heating of C_60_Cl_6_ solution in benzene and aliphatic alcohol leads to the partial elimination of chlorine atoms and the formation of 1,4-dialkoxy fullerene derivatives [12]. Notably, the reaction of C_60_Cl_6_ with alkali metal alcoholates leads to the formation of alkoxy fullerene derivatives C_60_(OR)_5_Cl with quite low yields (Figure 17).

**Figure 18:**
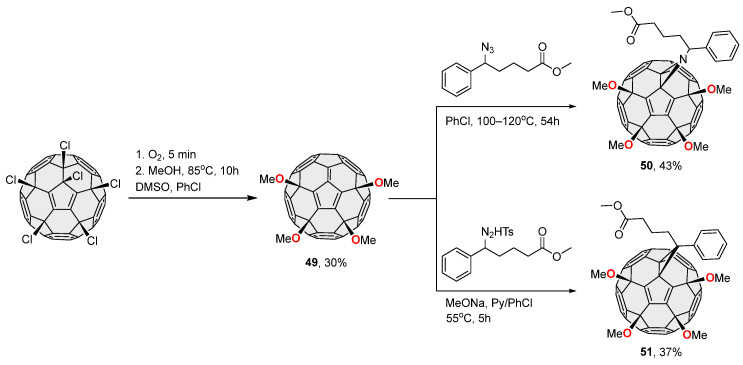
Synthesis and functionalization of the fullerene derivative C_60_(OCH_3_)_4_.

The reaction of C_60_Cl_6_ with methanol under aerobic conditions in the presence of dimethyl sulfoxide (DMSO) yields the methoxylated derivative C_60_(OCH_3_)_4_ (Figure 18) [88]. This transformation involves the elimination of two chlorine atoms, generating a reactive double bond that demonstrates remarkable selectivity in subsequent reactions with azides and tosylhydrazones.

**Figure 19:**
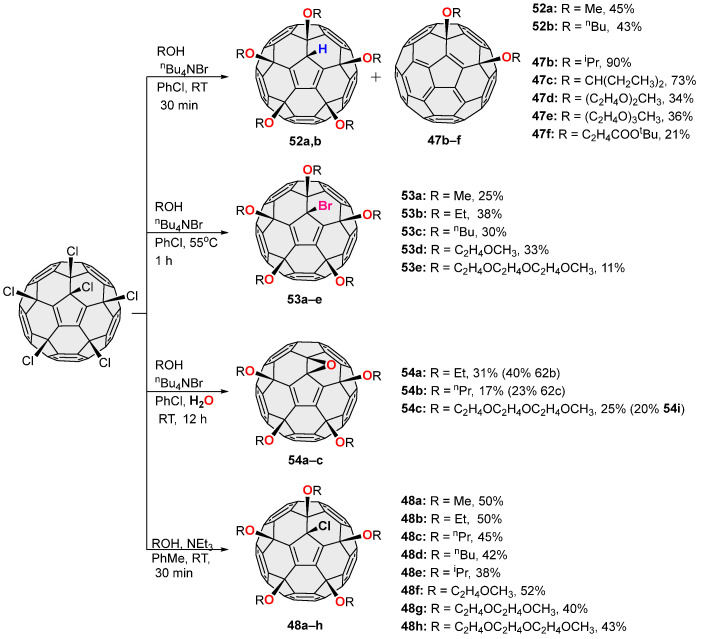
Synthetic routes to diverse alkoxy-functionalized fullerene derivatives.

Fullerene derivatives bearing five alkoxy residues attached to the fullerene cage can be obtained in fairly high yields using the reaction of chlorofullerene C_60_Cl_6_ with alcohols in the presence of tetrabutylammonium bromide or triethylamine. Depending on the reaction conditions, the developed method allows one to obtain alkoxy derivatives with the general formula C_60_(OR)_5_X (X=Cl, Br, H), C_60_(OR)_4_O or C_60_(OR)_2_ (Figure 19) [89].

**Figure 20:**
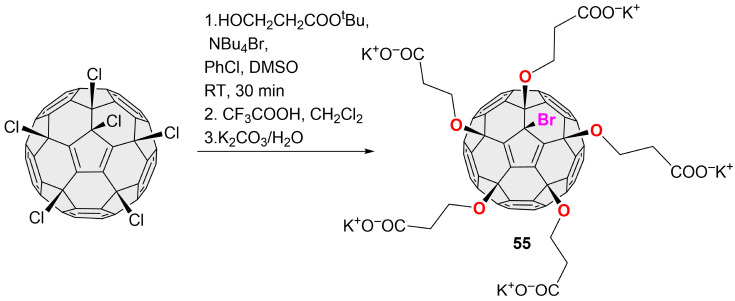
Synthesis of the fullerene derivative with the attached residues of hydroxy acids.

Although reactions of C_60_Cl_6_ with alcohols do not directly yield water-soluble fullerene derivatives, the observed reactivity patterns become particularly valuable when employing hydroxy acid esters instead of aliphatic alcohols. This approach enabled the synthesis of the first water-soluble polycarboxylic fullerene derivative featuring C-O bonds between the carbon cage and solubilizing groups (Figure 20) [89].

**Figure 21:**
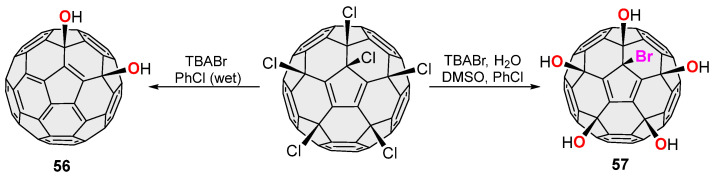
Synthesis of fullerenols 1,4-C_60_(OH)_2_ and C_60_(OH)_5_Br.

Fullerenols 1,4-C_60_(OH)_2_ and C_60_(OH)_5_Br were also synthesized via tetrabutylammonium bromide-promoted reactions of C_60_Cl_6_ with water [90]. Performing the synthesis in wet chlorobenzene produced 1,4-C_60_(OH)_2_ as the only isolable product along with substantial amounts of non-functionalized C_60_. However, the addition of DMSO as a co-solvent altered the reaction pathway, leading to the formation of C_60_(OH)_5_Br as the main product (Figure 21).

**Figure 22:**
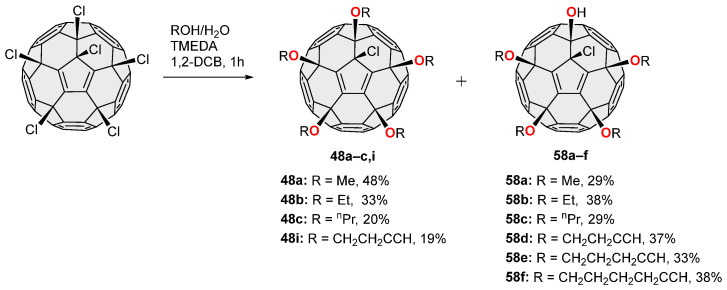
Preparation of alkoxy derivatives of fullerene in the presence of TMEDA.

In the presence of tetramethylethylenediamine (TMEDA), C_60_Cl_6_ also reacts with alcohols, with the formation of alkoxy derivatives C_60_(OR)_5_Cl and C_60_(OR)_4_(OH)Cl (Figure 22) [91].

**Figure 23:**
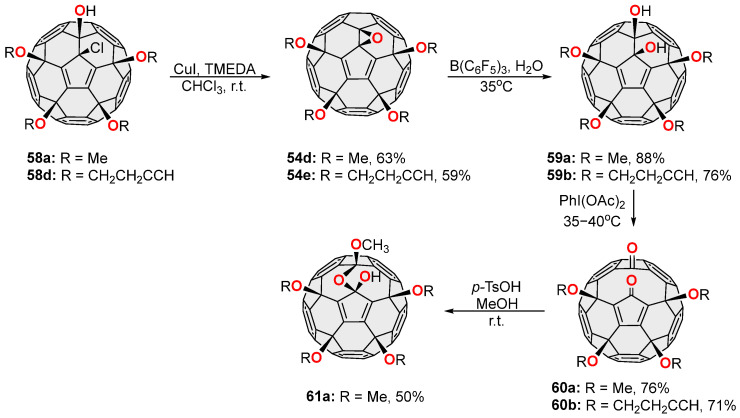
Synthesis of open-cage fullerene derivatives with alkoxy residues.

The obtained fullerene derivatives **58a**,**d** with four alcohol residues and a hydroxyl group can be used as precursors for the facile synthesis of open-cage fullerenes (Figure 23) [91].

Thus, the substitution of chlorine atoms in C_60_Cl_6_ with functionalized alcohols can also be considered an efficient pathway to water-soluble fullerenes with interesting molecular structures, including open-cage compounds. However, partial hydrolysis (the depletion of -OR groups with the formation of fullerenols) should be considered a limitation of this approach.

### 6.4. Reactions with Thiols and Mercapto Acids

**Figure 24:**
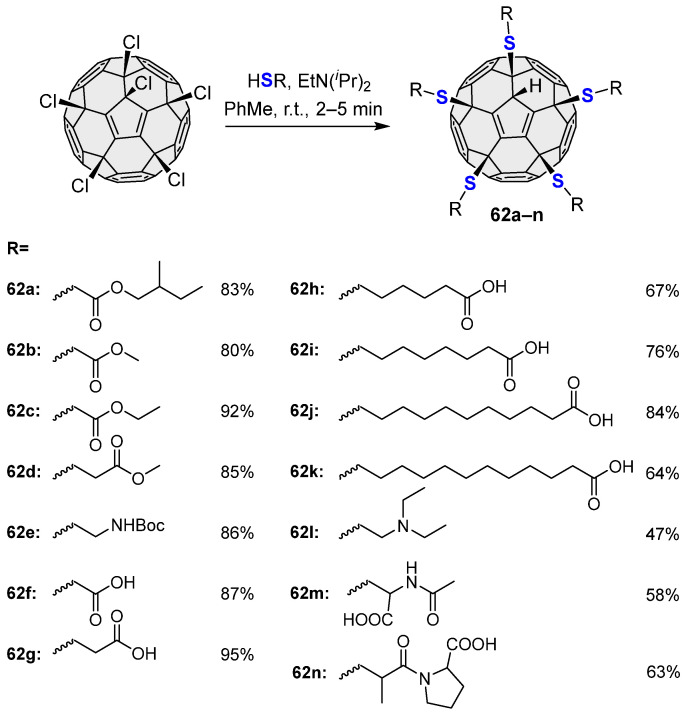
Reaction of C_60_Cl_6_ with functional thiols and mercapto acids.

Chlorofullerene C_60_Cl_6_ reacts readily with thiols and mercapto acids to yield derivatives containing five organic addends and a hydrogen atom attached to the fullerene cage. This method offers a fast, simple, and highly selective route to water-soluble fullerene derivatives. The transformation proceeds under exceptionally mild conditions (ambient temperature, aerobic atmosphere) using diisopropylethylamine as a base, yielding C_60_(SR)_5_H derivatives in excellent yields (47–95%). Notably, these products typically require no chromatographic purification, significantly simplifying the isolation process (Figure 24) [92].

**Figure 25:**
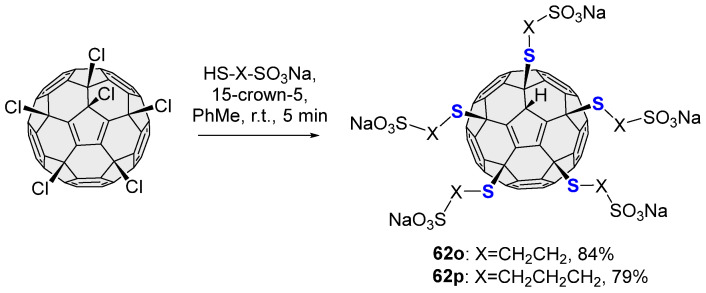
Synthesis of water-soluble fullerene derivatives using the reaction of C_60_Cl_6_ with sulfanylsulfonates.

Under slightly modified conditions (15-crown-5 ether, no base), C_60_Cl_6_ reacts with sodium sulfanylsulfonates to yield fullerene derivatives bearing sulfonate groups. Similar to their polycarboxylic analogs, compounds containing five SO_3_Na groups precipitate directly from the reaction mixture and can be isolated in pure form without chromatographic purification (Figure 25) [92].

**Figure 26:**
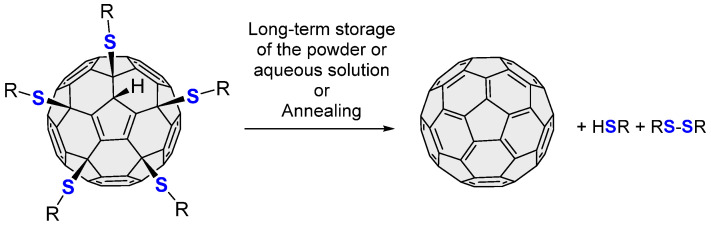
Thermal decomposition of fullerene derivatives with attached thiol residues.

The main limitation of this method is the poor long-term stability of the resulting compounds, both as solid powders and in aqueous solution. Freshly prepared C_60_(SR)_5_H derivatives gradually decompose to yield pristine C_60_, thiols, and disulfides (Figure 26). Although this instability makes them less suitable for biomedical applications, thermocleavable C_60_(SR)_5_H derivatives have found their application as precursors for the environmentally friendly fabrication of fullerene-based organic field-effect transistors [93].

### 6.5. Reactions with Phosphites

**Figure 27:**
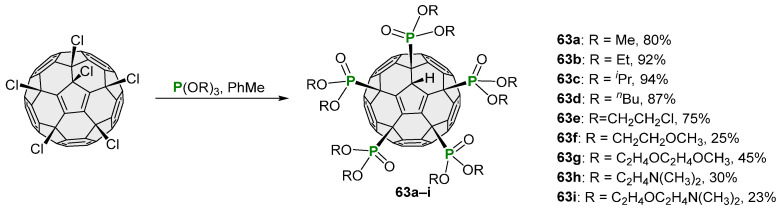
Reaction of C_60_Cl_6_ with trialkyl phosphites.

Similar to the classic Arbuzov (Michaelis–Arbuzov) reaction, where alkyl halides (RX) react with trialkyl phosphites (P(OR′)_3_ to form phosphonates (RP(O)(OR′)_2_) [94], chlorofullerene C_60_Cl_6_ undergoes an analogous transformation during the reaction with trialkyl phosphites. This reaction yields fullerene phosphonate derivatives C_60_[P(O)(OR)_2_]_5_H in good to excellent yields (23–94%) (Figure 27) [95].

**Figure 28:**
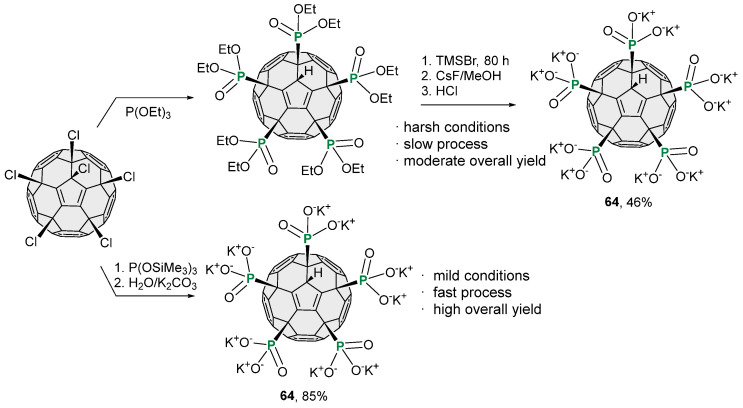
Strategies for the synthesis of phosphonate water-soluble fullerene derivatives.

Notably, fullerene derivatives bearing some alkyl phosphonate groups exhibit good water solubility. Although the phosphonate moieties (-PO(OR)_2_) can be hydrolyzed to phosphonic acid groups (-PO(OH)_2_) using bromotrimethylsilane to yield anionic water-soluble compounds and enhance solubility (Figure 28), this deprotection step proceeds under harsh conditions.

To avoid harsh deprotection conditions, a more direct approach based on the C_60_Cl_6_ reaction with tris(trimethylsilyl) phosphite (P(OTMS)_3_) was proposed. TMS groups in the structure of the fullerene derivative are easily depleted via water/acid treatment [96].

### 6.6. Reactions with C-H Acids and Enol Silyl Ester

**Figure 29:**
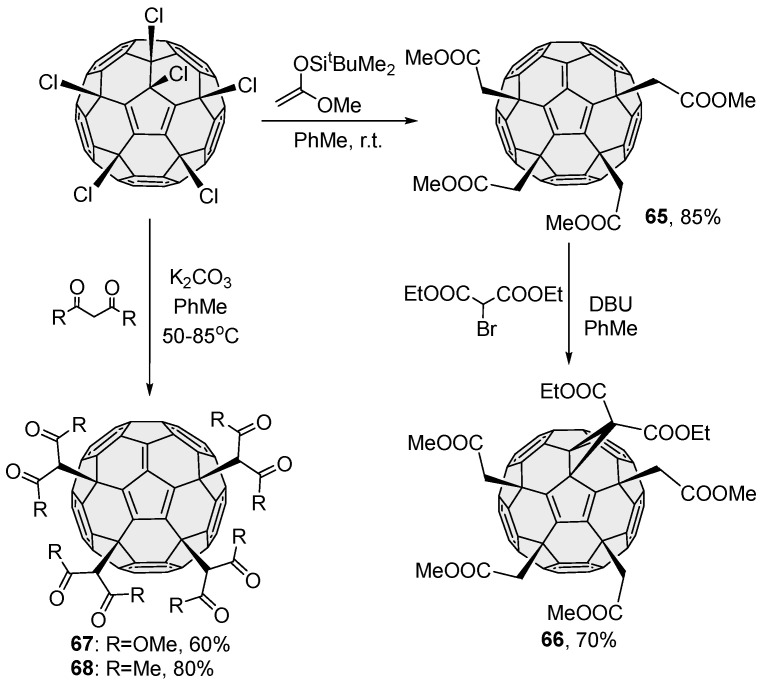
Reaction of C_60_Cl_6_ with CH-acids and enol silyl ester.

Chlorofullerene C_60_Cl_6_ also regioselectively reacts with CH-acids and enol silyl ester (Figure 29) [97]. Both reactions lead to the formation of *C_s_*-symmetrical compounds with four aliphatic addends that can be further selectively converted to fullerene derivatives with a cyclopentadienyl-type addition pattern, similarly to C_60_(OCH_3_)_4_ derivatives, shown in Figure 18.

### 6.7. Reactions with Aromatic Compounds

As demonstrated above, halogen atoms on the fullerene cage can be easily substituted with a broad range of organic residues attached to the fullerene cage via C–N, C–P, C–S and C–O bonds. Although most of these reactions proceed in one step and with nearly quantitative yields, many of the resulting fullerene derivatives appeared to be not fully stable if stored as aqueous solutions.

In contrast, water-soluble fullerene derivatives with organic addends attached to the carbon cage via C–C bonds are inherently stable, can be stored in aqueous media, and, therefore, appear to be one of the most promising types of water-soluble fullerene derivatives for biomedical applications.

Several approaches to form C-C bonds with a fullerene cage via the substitution of chlorine atoms have been reported, including C_60_Cl_6_ reactions with organolithium compounds [98,99,100], allyltrimethylsilane [101], arylacetylenes [102], and organic cyanides [103].

**Figure 30:**
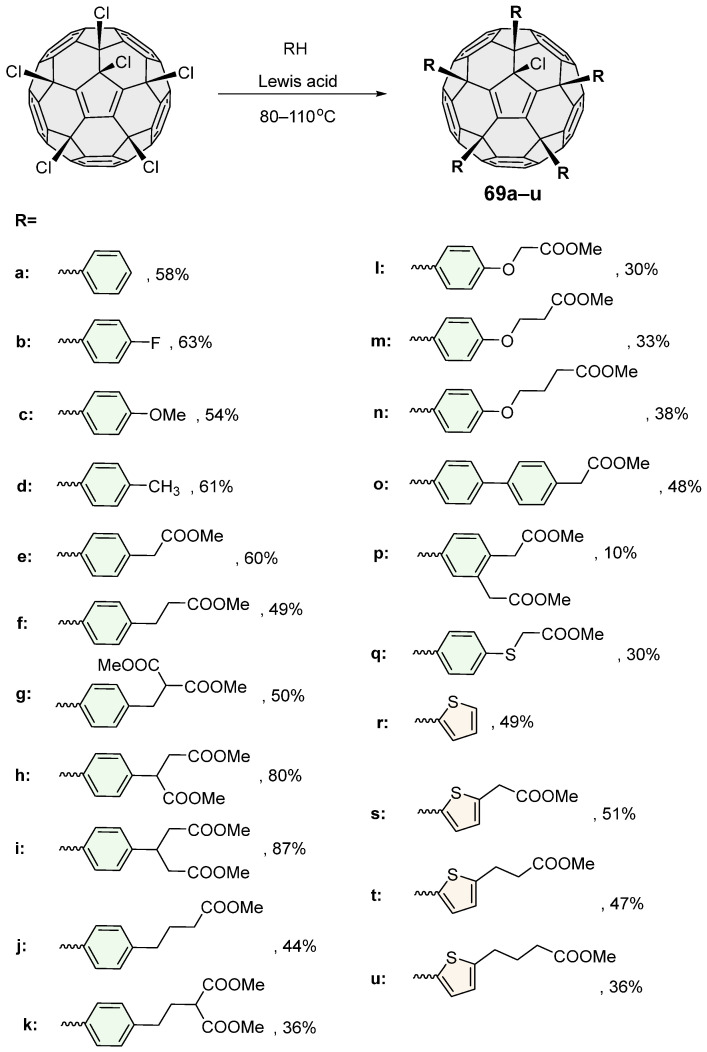
Friedel–Crafts arylation reaction of chlorofullerene C_60_Cl_6_.

One of the most versatile approaches to creating water-soluble compounds is based on Friedel–Crafts arylation with benzene- or thiophene-based compounds. C_60_Cl_6_ reacts with aromatic compounds via the electrophilic substitution reaction in the aromatic ring. The substitution of chlorine atoms by aryl or heteroaryl residues (Ar) occurs directly in the aromatic hydrocarbon medium or in a suitable inert solvent (Figure 30) [9,104]. When substituted benzenes are used as substrates, the reaction predominantly yields para-substituted products with the general structure C_60_Ar_5_X (where X = Cl or H).

**Figure 31:**
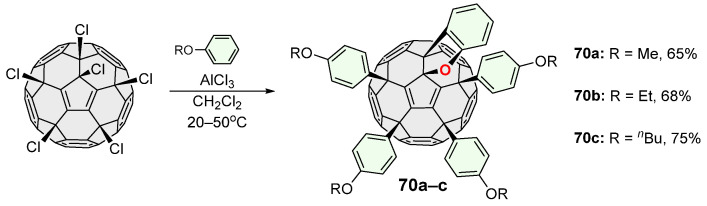
Friedel–Crafts arylation reaction of chlorofullerene C_60_Cl_6_ with phenol ethers.

Notably, when phenol or hydroquinone derivatives are used in arylation reactions, several products with non-typical structures can be formed (Figure 31) [105].

**Figure 32:**
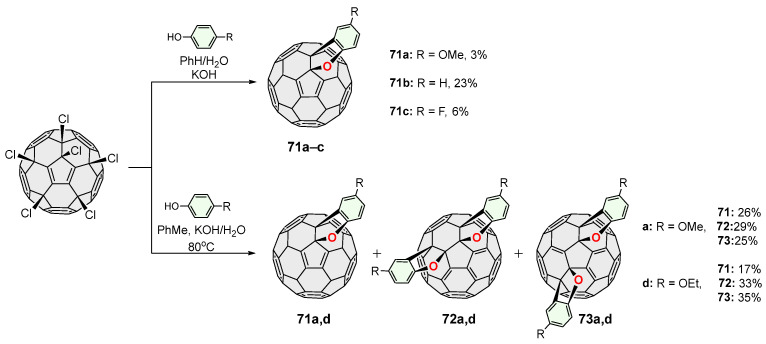
Interaction of C_60_Cl_6_ with phenols in the presence of a base.

In particular, the reaction of C_60_Cl_6_ with alkyl phenols and alkyl phenyl ethers allows us to obtain non-classical products with a dihydrobenzofuran fragment fused to the cage (Figure 32) [106].

**Figure 33:**
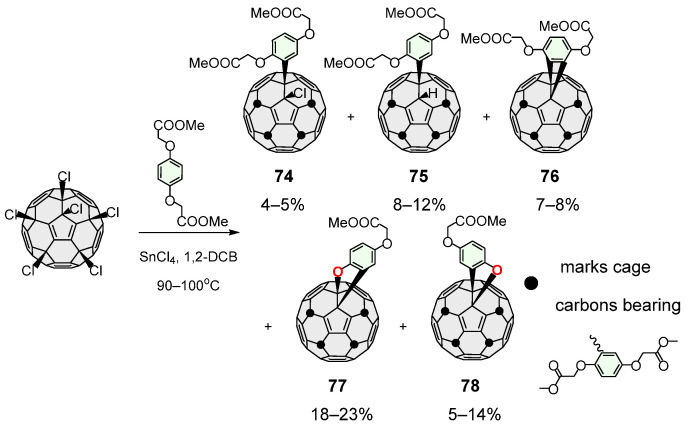
The reaction of C_60_Cl_6_ with hydroquinone ether leading to the formation of a mixture of products with atypical structure.

The arylation of chlorofullerene C_60_Cl_6_ with dimethyl-2,2′-(1,4-phenylenebis(oxy))-diacetate, catalyzed by SnCl_4_, leads to the formation of a mixture of five different products, which were isolated using preparative HPLC. In addition to the two classical products of the reaction C_60_Ar_5_X (X=H, Cl), two derivatives with dihydrobenzofuran fragments and one compound with a cyclobutane fragment were also isolated (Figure 33) [107].

Water-soluble polycarboxylic derivatives can be synthesized via an arylation reaction using ArH substrates containing ester groups. The subsequent hydrolysis of these esters to carboxylic acids, followed by treatment with K_2_CO_3_ or Na_2_CO_3_, yields the target water-soluble salts.

**Figure 34:**
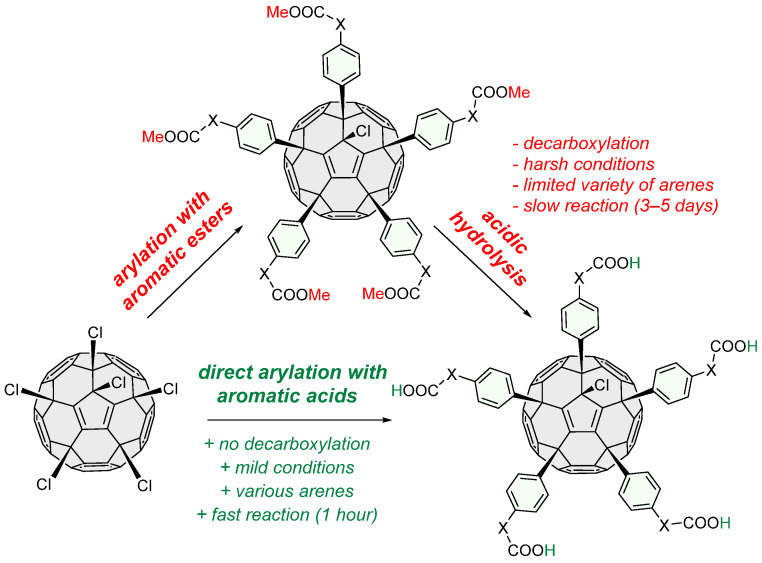
Strategies for the preparation of water-soluble fullerene derivatives with aromatic addends.

Recently, a more efficient approach based on the direct arylation of C_60_Cl_6_ with unprotected carboxylic acids was developed (Figure 34) [108]. This method significantly reduces the number of synthetic steps and the overall reaction time. Moreover, since it does not include an acidic hydrolysis step, this strategy is suitable for the synthesis of fullerene–amino acid conjugates.

However, the scope of this method is severely limited to those substrates that could tolerate the rather harsh conditions of the Friedel–Crafts arylation reaction (high temperature, the presence of a Lewis acid).

**Figure 35:**
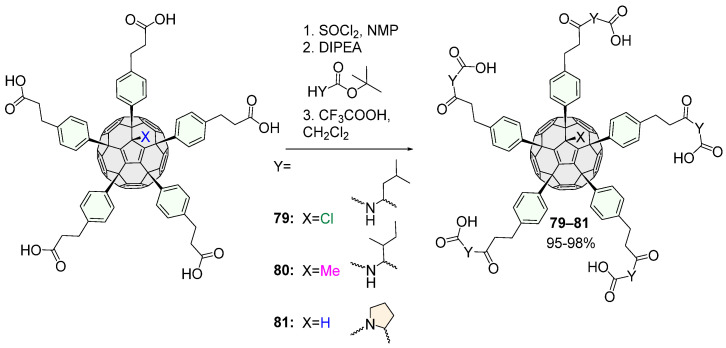
Acylation of tert-butyl esters of amino acids with fullerene-based chloroanhydrides formed in situ.

That is why an alternative approach to the synthesis of fullerene derivatives loaded with amino acid residues through the acylation of amino acid tert-butyl esters with in situ-formed fullerene-based chloroanhydride was proposed (Figure 35) [109]. The optimized conditions of this reaction do not impose a limitation on the structure of the amino acid and allow the facile synthesis of numerous adducts starting from a single fullerene-based precursor.

Since the sixth chlorine atom attached to the fullerene cage usually cannot be substituted with a large aromatic residue due to steric hindrances, the possibility of its substitution with smaller functional groups and various rearrangements was investigated.

**Figure 36:**
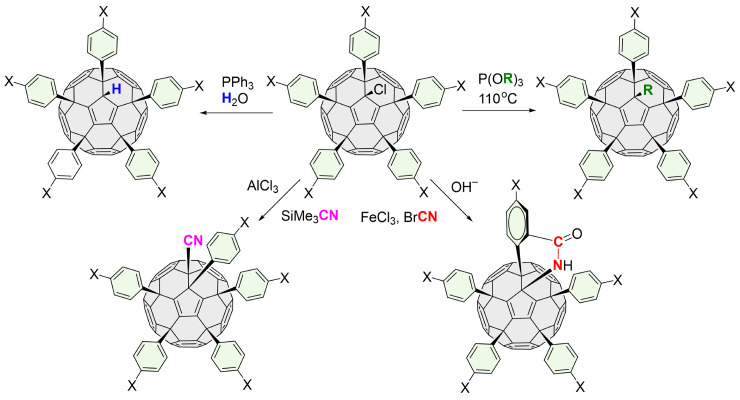
Chemical transformations of fullerene derivatives C_60_Ar_5_Cl.

One of the first discovered substitution reactions was the interaction of pentaarylated fullerene derivative C_60_Ar_5_Cl with water and triphenylphosphine with the formation of C_60_Ar_5_H adducts [6]. The substitution of the Cl atom with alkyl residue is possible if the fullerene derivative C_60_Ar_5_Cl is treated with trialkylphosphite under “abnormal” Arbuzov reaction conditions (Figure 36) [110].

When C_60_Ph_5_Cl interacts with the Lewis acid, heterolytic dissociation of the C-Cl bond leads to the formation of the antiaromatic *C*_5*v*_-symmetric carbocation [C_60_Ph_5_]^+^. One of the phenyl groups undergoes a 1,2-shift, forming a more stable carbocation *C_s_*-[C_60_Ph_5_]^+^. The position of the positive charge in the *C_s_*-[C_60_Ph_5_]^+^ cation was confirmed using the isolation and characterization of the fullerene derivative *C_s_*-[C_60_Ph_5_]CN obtained via the treatment of *C_s_*-[C_60_Ph_5_]^+^ with TMSCN reagent; however, this result was only mentioned in a review paper (Figure 36) [111].

The reaction of C_60_Ph_5_Cl with BrCN in the presence of FeCl_3_ results in the substitution of the chlorine atom followed by the substitution of the hydrogen atom of the adjacent phenyl ring (Figure 36). The treatment of the product with alkali results in the formation of the 1-isoquinolone derivative [112].

**Figure 37:**
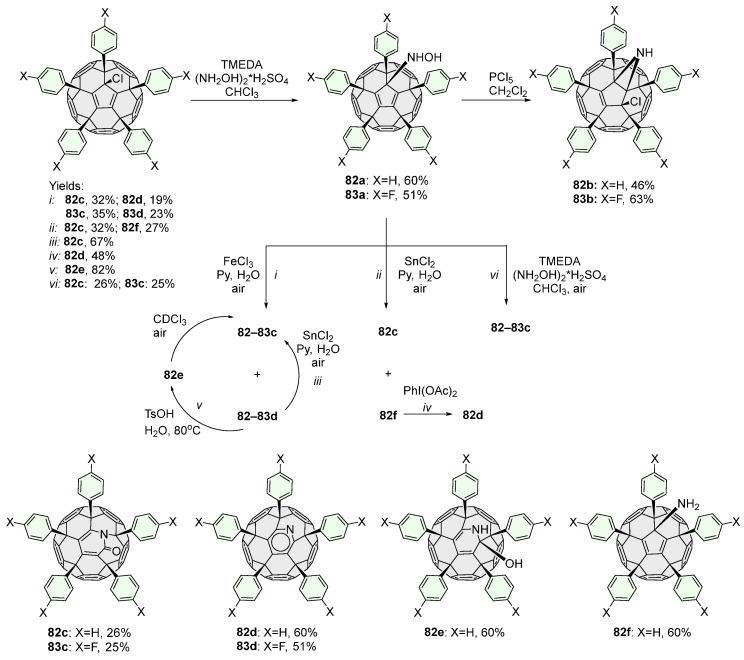
Synthesis and transformation of fullerene derivatives C_60_Ar_5_(NHOH).

The treatment of fullerene derivatives C_60_Ar_5_Cl with hydroxylammonium sulfate and TMEDA (Figure 37) yields C_60_Ar_5_(NHOH) derivatives (**82**–**83a**) [113]. The subsequent reaction of **82**–**83a** with phosphorus pentachloride produces aziridine-containing derivatives **82**–**83b**, which feature both an aziridine ring and a chlorine substituent.

Unexpectedly, attempts to replace the chlorine atom in **82**–**83b** with an –NHOH group (using hydroxylammonium sulfate/TMEDA) did not yield the anticipated products. Instead, this transformation led to the formation of derivatives **82**–**83c** containing a pyridinone fragment.

Further reactivity studies revealed that the treatment of **82**–**83b** with Lewis acids (FeCl_3_ or SnCl_2_) results in partial conversion to **82**–**83c**, along with the formation of unique derivatives **82**–**83d**–**f**.

**Figure 38:**
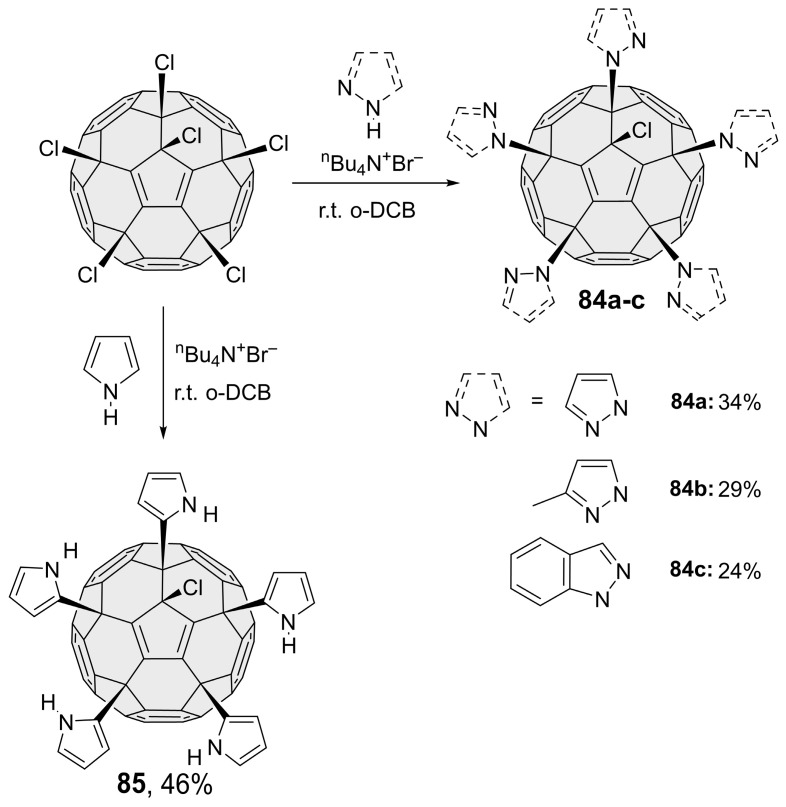
Reactions of C_60_Cl_6_ with pyrroles and pyrazoles.

Gan et al. demonstrated that chlorofullerene C_60_Cl_6_ reacts with pyrroles and pyrazines, forming products with five residues attached via either C-C or C-N bonds (Figure 38). Pyrroles and pyrazines bearing carboxylic or other polar groups can potentially be utilized for the synthesis of water-soluble derivatives [114].

Thus, chlorofullerenes, particularly C_60_Cl_6_, serve as highly reactive intermediates for the efficient synthesis of water-soluble derivatives. Their multiple chlorine substituents enable diverse functionalization pathways, facilitating attachment of various functional groups with versatile addition patterns.

### 6.8. Fluorinated Fullerenes as Precursors for Water-Soluble Fullerene Derivatives

It should be noted that efficient gram-scale synthetic methods are currently available only for the halogenated fullerenes C_60_F_18_, C_60_Cl_6_, C_70_Cl_8_ and C_70_Cl_10_. Similarly to C_60_Cl_6_, C_60_F_18_ also exhibits high selectivity in reactions with nucleophiles.

**Figure 39:**
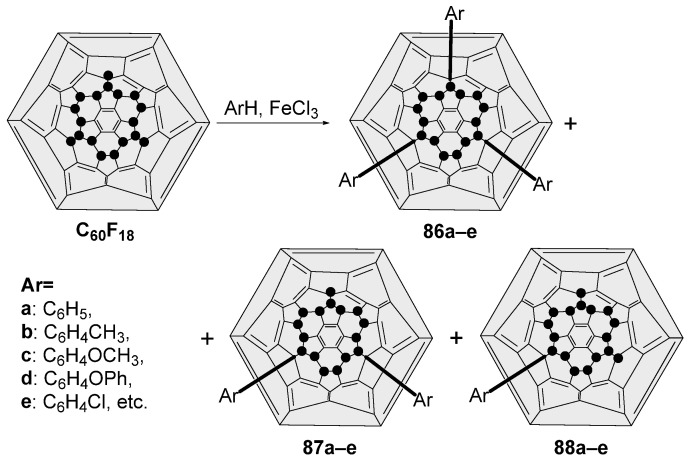
Arylation of fluorofullerene C_60_F_18_.

The reactivity of fluorofullerene C_60_F_18_ was actively investigated by Prof. R. Taylor and colleagues. C_60_F_18_ can be prepared using the reaction between C_60_ and potassium hexafluoroplatinate under elevated temperatures [115]. They revealed that the fluorine atoms in C_60_F_18_ can be substituted with aryl groups during the reaction with ArH in the presence of FeCl_3_, yielding C_60_F_15_Ar_3_ (Figure 39) [116]. This reaction holds significant potential for the synthesis of water-soluble fullerene derivatives if the Ar substituent incorporates two or more polar hydrophilic groups, such as a carboxylic group.

**Figure 40:**
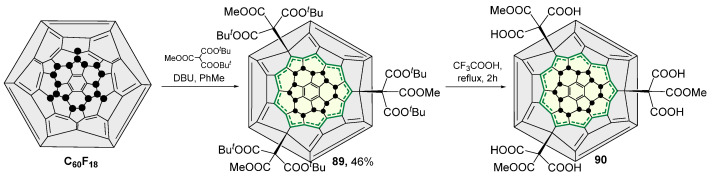
Synthesis of water-soluble trannulene from C_60_F_18_.

The reaction of C_60_F_18_ with carbanionic nucleophiles follows a distinctive S_N_2″ mechanism, leading to the formation of trannulenes with the general formula C_60_F_15_R_3_. This S_N_2″ pathway is exclusive to the chemistry of fluorinated fullerenes and involves nucleophilic attack at a δ-carbon atom, triggering a shift in two conjugated double bonds and subsequent elimination of fluorine (Figure 40) [117]. Several unique water-soluble trannulenes were synthesized using this approach.

**Figure 41:**
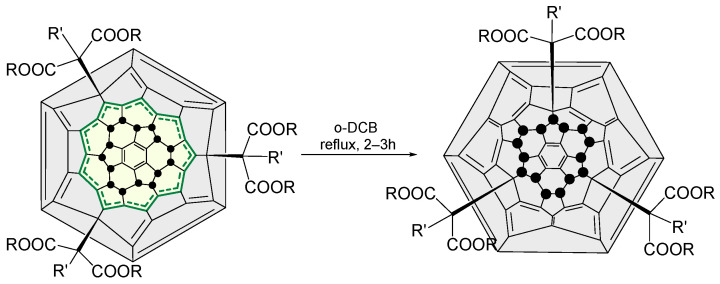
Synthesis of triumphenes from trannulenes.

The 18-membered aromatic macrocycle provides trannulenes with high stability and unique optoelectronic properties. However, they were later found to undergo thermal isomerization to triumphenes with high yields (70–95%) [118]. Thus, thermal isomerization of trannulenes offers a pathway to the synthesis of new triumphenes with aliphatic addends, which cannot be obtained directly from fluorofullerene C_60_F_18_ (Figure 41). Further hydrolysis of ester groups can lead to the formation of water-soluble triumphenes with aliphatic addends.

## 7. Fullerene Derivatives with Several Types of Addends

Since even the introduction of several identical addends to the fullerene cage often leads to the formation of isomeric mixtures, the regioselective synthesis of fullerene derivatives bearing several types of addends represents an even more complex synthetic challenge.

While several examples of statistical synthesis for fullerene derivatives bearing two distinct functional groups have been reported [119], these methods suffer from significant limitations. The lack of regioselectivity in these reactions typically results in complex product mixtures, making the isolation of individual compounds challenging even with preparative HPLC.

**Figure 42:**
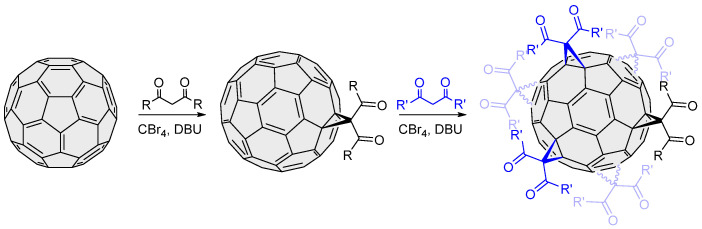
Sequential Bingel–Hirsch reactions for the regioselective synthesis of polyfunctionalized fullerenes.

Among the approaches that allow us to introduce two types of addends on the fullerene cage in a regioselective manner, sequential Bingel–Hirsch reactions (Figure 42) can be given as an example [4]. First, the mono-adduct is obtained in good yield by carefully controlling the stoichiometry of the malonate. In the second step, adding an excess of another 1,3-dicarbonyl compound completes the octahedral addition pattern.

**Figure 43:**
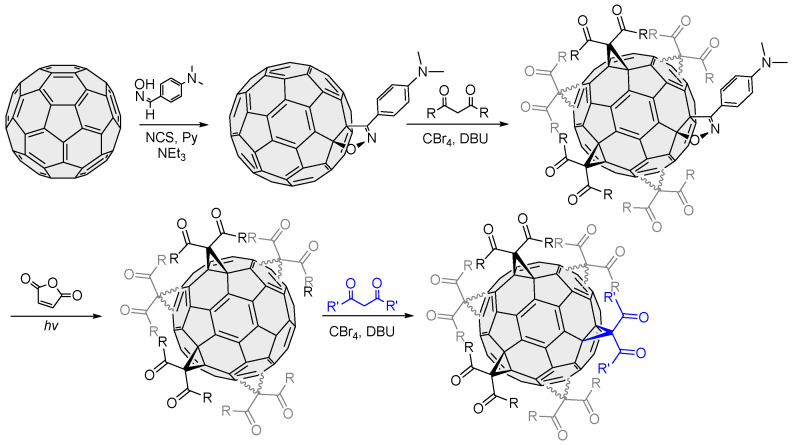
Synthesis of fullerene derivatives with two types of addends using retro dipolar cycloaddition of nitrile oxides.

The malonate addition sequence can be reversed to 5 + 1 if the strategy based on the retro dipolar cycloaddition of nitrile oxides to the fullerene cage is applied (Figure 43) [120].

**Figure 44:**
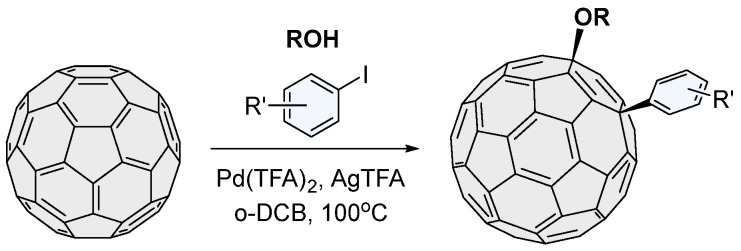
Palladium-catalyzed three-component alkoxyarylation of C_60_.

Fullerene derivatives featuring two different addends can also be prepared via palladium-catalyzed three-component C_60_ alkoxyarylation (Figure 44) [121]. However, this approach yields only 1,4-adducts with a low degree of functionalization, precluding its application for the synthesis of water-soluble fullerene derivatives.

**Figure 45:**
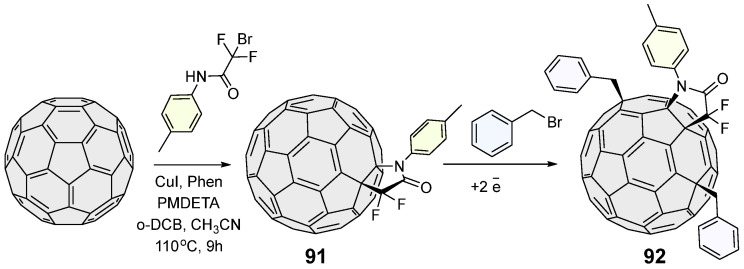
Cu(I)-catalyzed synthesis of fullerene-fused lactams and further electrochemical functionalization.

Heteroannulation of C_60_ with α-bromo acetamides or anilides followed by electrochemical functionalization also yields fullerene derivatives with both benzyl-type addends and a fused lactam moiety (Figure 45) [122].

**Figure 46:**
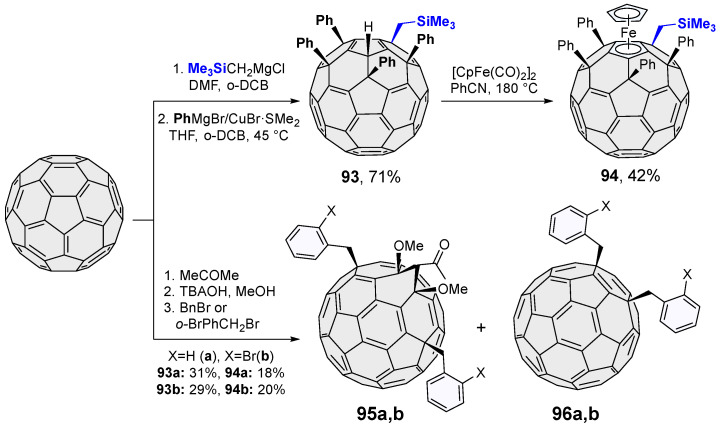
Regioselective synthesis of fullerene derivative with three types of addends.

The synthesis of fullerene derivatives bearing three types of functional addends on the cage is so far represented by the reaction of C_60_Ph_4_(CH_2_SiMe_3_)H with [CpFe(CO)_2_]_2_ (Figure 46) [123] and the reaction of C_60_ with acetone under basic conditions coupled with intermediate quenching with benzylbromide derivatives [124].

Most of these reactions have a limited synthetic scope, and, with a couple of exceptions, they can hardly be used for the introduction of the solubilizing polar groups required for the synthesis of water-soluble compounds with tailored biological properties.

**Figure 47:**
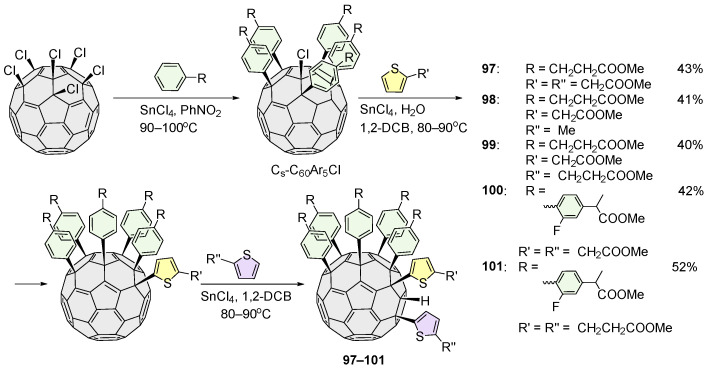
Synthesis of novel fullerene derivatives C_1_-C_60_Ar_5_Th′_2_H and *C*_1_-C_60_Ar_5_Th′Th″H.

Water-soluble fullerene derivatives with two or more types of addends were synthesized either via sequential cyclopropanation, as discussed in Section 2, or via the approach based on C_60_Ar_5_Cl reactions with substituted thiophenes, which enables the substitution of Cl with thiophene residues and the formation of the novel family of *C*_1_-symmetrical C_60_ fullerene derivatives with six functional addends C_60_Ar_5_Th. Fullerene derivatives *C*_1_-C_60_Ar_5_Th’ also react with another equivalent of substituted thiophene Th″H, leading to the formation of the unique family of water-soluble compounds *C*_1_-C_60_Ar_5_Th′Th″H with three types of functional addends (Figure 47) [125].

## 8. Biological Activity of Individual Water-Soluble Fullerene Derivatives

Despite two decades of active development of synthetic methods for individual polyfunctionalized fullerene derivatives, most studies on the biological activity of water-soluble fullerene-based compounds have been conducted using complex, inseparable mixtures of fullerenols and related substances. However, when the goal is mechanistic understanding, therapeutic development, or regulatory approval, isomerically pure fullerene compounds should be used. Mixed-structure materials are acceptable only for early-stage exploratory work, non-clinical uses, or bulk applications—situations where high reproducibility and detailed mechanistic knowledge are not critical.

**Figure 48:**
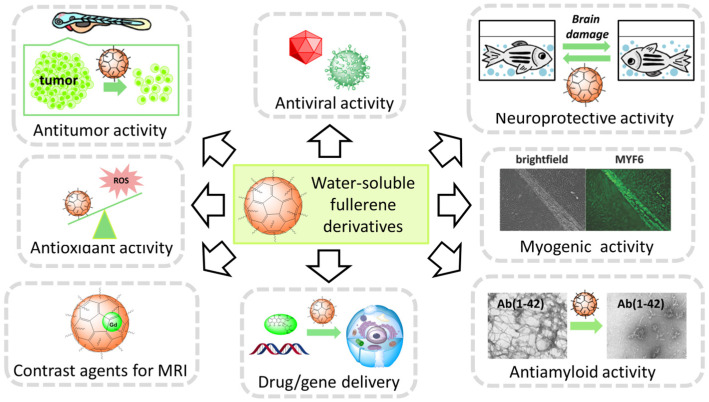
Biological activity of water-soluble fullerene derivatives: general research directions.

Regardless their rather limited synthetic accessibility, individual water-soluble fullerenes were investigated both in vitro and in vivo. Key research directions include antiviral and antimicrobial activity, antitumor, antioxidant and antiischemic properties, neuroprotection, radiation protection, drug delivery systems, bioimaging, and stem cell modulation (Figure 48).

### 8.1. Fullerene–Cell Interactions and Localization

**Figure 49:**
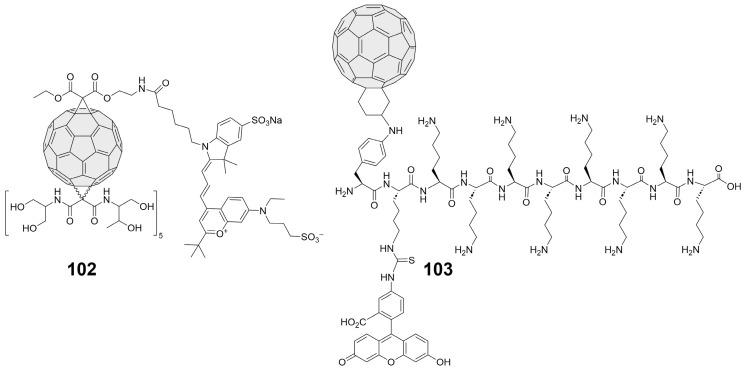
Individual fullerene derivative with endocytotic uptake pathway.

Several studies implicated energy-dependent endocytotic pathways in the uptake of functionalized fullerenes. For example, Curley et al. have reported this for the individual water-soluble compound **102**, which after uptake was preferentially localized in the cell nucleus (Figure 49) [126]. The uptake of an individual water-soluble fullerene-substituted phenylalanine poly-lysine derivative with an FITC label **103** involves a complex, ATP-dependent endocytic mechanism mediated by caveolae/lipid rafts, F-actin, and microtubules, likely proceeding through a scavenger receptor-specific pathway [127].

Malonic acid-derivatized mono-adduct C_60_(COOH)_2_ and hexa-adduct C_60_(COOH)_6_ localize predominantly to the mitochondria [128,129], whereas the isomerically pure bis-adduct C_60_(C(COOH)_2_)_2_ localizes to lysosomes [130].

The protein corona fundamentally redefines the biological identity, cellular interactions, and in vivo fate of nanoparticles. The complex interactions between fullerenes and proteins fall into three primary subgroups: π–π stacking involving fullerene sp^2^ carbons and aromatic amino acid residues, van der Waals contacts between the C_60_ cage and the protein surface, and hydrophobic interactions (nonpolar solvation) [131]. Fullerenes can interact with serum proteins so that a protein corona is formed around the nanoparticles, but the detailed composition of corona is still unknown [132].

Research has shown that protein binding with the C_60_ nanocomplex did not change the charge properties of the protein. The proteins induced the aggregation of the C_60_ nanocomplex [133]. Bai et al. have recently developed an artificial intelligence model for the accurate prediction of protein targets of individual isomerically pure amphiphilic fullerene derivatives, which will be extremely promising for the rational design of fullerene-based therapeutics, drug discovery, and precision nanomedicine [134].

### 8.2. Acute Toxicity

The pharmacokinetic behavior and toxicological effects of fullerenes are highly influenced by their route of administration and are closely related to their functionalization [135]. Extensive studies confirm that pristine C_60_ exhibits no acute or sub-acute toxicity across diverse biological systems, including bacteria, fungi, human leukocytes, and model organisms such as Drosophila, mice, rats, and guinea pigs [136].

Short-term in vitro studies have revealed that high concentrations of unmodified C_60_ can induce rapid cell membrane damage and apoptosis in certain cell lines [137], but this effect is mitigated when fullerenes are properly solubilized. Thus, early claims of the toxicity of fullerene derivatives can be associated with the difficulty of their removal from organisms and the use of co-solvents, such as tetrahydrofuran, for solubilization.

**Figure 50:**
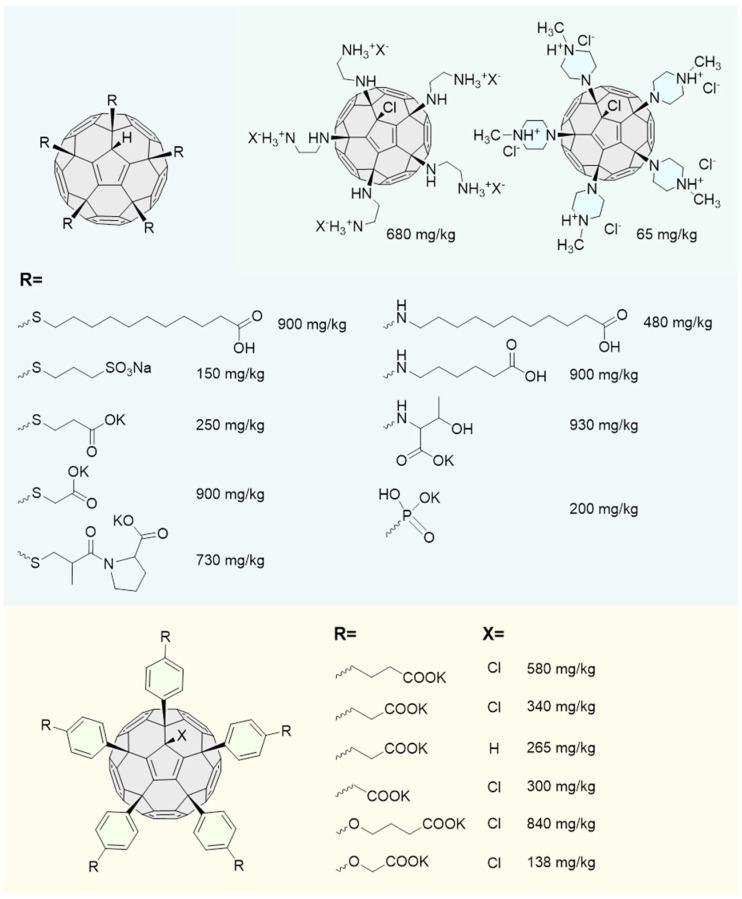
Acute toxicity of individual water-soluble fullerene derivatives.

Acute toxicity in mice was investigated for several individual water-soluble fullerenes, including arylated compounds, compounds with attached residues of aminoacids, thioacids, etc. (Figure 50) [138]. Most of them demonstrated low toxicity with LD_50_ values exceeding 300 mg/kg.

### 8.3. Antiviral Properties

The active investigation of their anti-HIV activity began in 1993 when Shinazi and colleagues [139] reported the anti-HIV activity of a water-soluble fullerene derivative in infected cells, while Friedman et al. [140] proposed allosteric HIV-1 protease inhibition as a possible mechanism of antiviral activity. According to molecular docking results, C_60_ bis(phenethylaminosuccinate) was able to interact with the active site of HIV-1 protease, and these theoretical findings were supported by cell-free HIV-1 protease inhibition assays [140]. Further works by Toniolo et al. [141], Friedman et al. [142], and Strom et al. [143] revealed a wide range of fullerene derivatives able to inhibit the catalytic activity of HIV-1 protease in micro- and nanomolar concentrations in cell-free experiments. Marchesan et al. showed that five isomeric bis-fulleropyrrolidinium salts **29**–**33** are inhibitors of HIV-1 and HIV-2, finding that the positions of the side chains influence antiviral activity, and *trans*- isomers of the fullerene bis-adducts are more active than the *cis*- one [71]. The same group also investigated the anti-HIV properties of pegylated polycationic fullerene derivatives **34**–**36** and showed that positive charges near to the C_60_ backbone could increase potency, while longer solubilizing chains seem to induce cytotoxicity [72]. Polycarboxylic fullerene derivatives derived from chlorofullerenes C_60_Cl_6_ and C_70_Cl_8_ were investigated as broad-spectrum antivirals. The most efficient compounds **104a**–**c** inhibited HIV at concentrations of 150–350 nM.

A water-soluble fullerene conjugate with cyclodextrin **5** was shown to be an efficient hepatitis C virus entry inhibitor [49].

**Figure 51:**
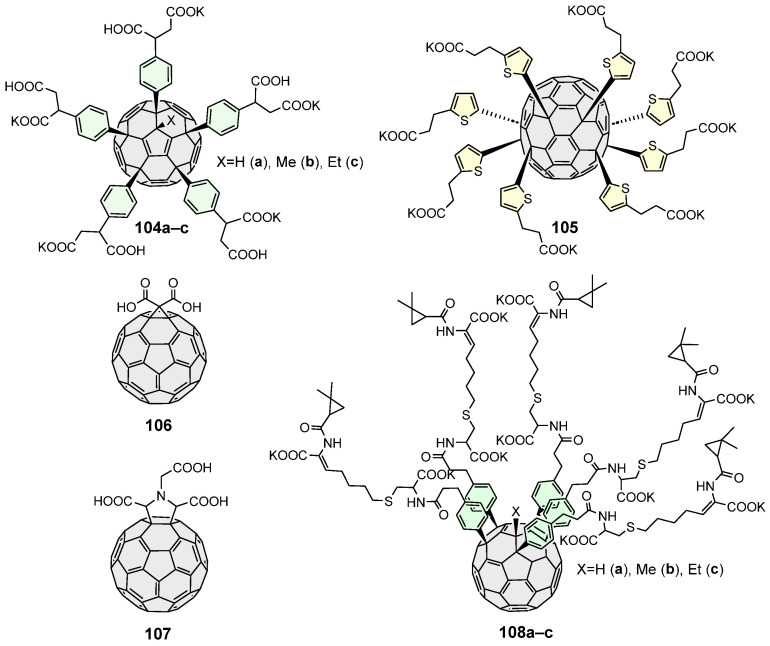
Polycarboxylic fullerene derivatives demonstrating promising antiviral properties.

The most promising inhibition of herpes simplex virus (HSV) was demonstrated for the compounds incorporating thiophene residues. For example, compound **105** demonstrated a selectivity index (SI) of 40,380 (Figure 51) [144].

Water-soluble conjugates with cyclodextrin **6** and **7** efficiently inhibited the Influenza A (H1N1) virus in vitro in micromolar concentrations [49]. Among a wide range of pentacarboxylic fullerene derivatives, maximum virus-inhibiting activity and minimum toxicity were found for the compound containing residues of salts of 2-amino-3-cyclopropylpropanoic acid (CC_50_ > 300 µg/mL, IC_50_ = 4.73 µg/mL, SI = 64) [145].

Several recent reviews have identified fullerenes as a promising platform for developing next-generation antivirals against SARS-CoV-2, particularly through the inhibition of viral enzymes [146,147,148]. Computational studies have largely focused on either pristine C_60_ and C_70_ fullerenes [149,150] or their functionalized derivatives [151,152,153,154], probing interactions with a range of SARS-CoV-2 targets. These include the proteases Mpro (3CLpro) and PLpro, RNA-dependent RNA polymerase, the spike glycoprotein and its human receptor hACE2, the nucleocapsid protein’s RNA-binding domain, and even combinations of fullerene derivatives with anti-COVID drugs like favipiravir and chloroquine. Petitjean used atomic force microscopy to screen multivalent fullerene-based glycoclusters capable of disrupting the interaction between the SARS-CoV-2 spike protein and the glycan 9-O-acetylated sialic acid (9-AcSA), a molecule believed to be critical for viral attachment to host cells [155]. Despite strong interest in the antiviral potential of fullerene derivatives against coronaviruses—SARS-CoV-2 in particular—only few experimental studies have so far assessed their activity using either viral enzymes or infected cells. In one, Katagishi et al. tested several fullerene derivatives for their ability to inhibit the SARS-CoV-2 main protease [156]. A polycarboxylic malonic acid-type derivative **106** and pyrrolidino [60]fullerene derivative **107** emerged as the most effective, achieving 50% protease inhibition at just 0.53 and 0.98 μM. Another study introduced polycarboxylic fullerene derivatives **108a**–**c** (Figure 50) with cilastatin-based addends representing promising antiviral agents for COVID-19 treatment (IC_50_ = 390–620 nM) [157].

In vitro screening of the individual fullerene derivative C_60_(P(O)(OK)_2_)_5_H showed that it demonstrates broad-spectrum antiviral activity against a panel of ten distinct viruses, including Varicella zoster virus, cytomegalovirus, HSV, cowpox virus, feline coronavirus, feline herpes virus, etc. [96].

Glycoconjugates **18**–**20** with mannose residues attached to a fullerene cage synthesized by Prof. Nierengarten and colleagues effectively inhibited the DC-SIGN-dependent (dendritic cell-specific intercellular adhesion molecule-3-grabbing nonintegrin) entry of Ebola virus envelope glycoprotein EboGP pseudotyped viral particles into cells. Most of the conjugates effectively bound to the DC-SIGN receptor, blocking its interaction with viral glycoproteins at a concentration as low as 0.667 nM [60].

**Figure 52:**
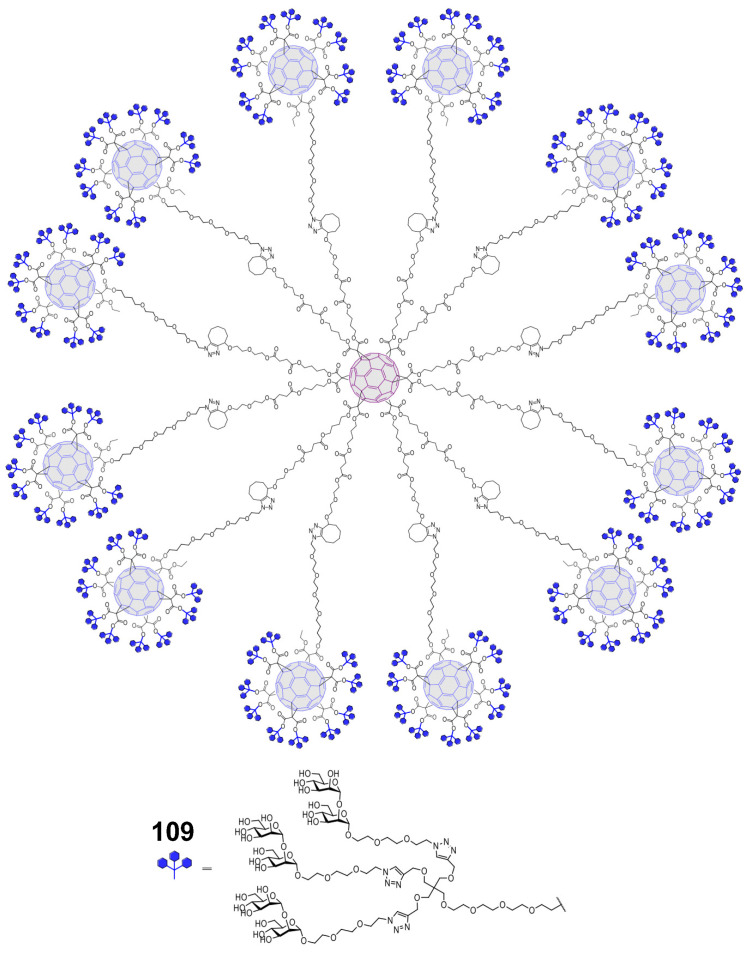
Tridecafullerene appended with 360 1,2-mannobioside units using the SPAAC synthetic pathway. Figure adapted from ref. [61].

Cell entry of Zika (ZIKV) and dengue viruses (DENVs) also involves several receptors, including DC-SIGN. Glycoconjugates containing 36–360 mannose residues in their structure were obtained via the strain-promoted cycloaddition of azides to alkynes (SPAAC) strategy. These compounds also efficiently blocked the DC-SIGN receptor through multivalent interactions. They demonstrated remarkable antiviral activity against Zika and dengue viruses with 50% inhibitory doses (IC_50_) in the nano- and picomolar ranges. Tridecafullerene **109** (Figure 52) appended with 360 1,2-mannobioside showed the best IC_50_ values for both the ZIKV (67 pM) and the DENV (35 pM) [61]. Considering that no vaccine or specific antiviral drug has yet been approved for these viral tropical fevers, the obtained results look promising and can be used for the development of effective antiviral drugs.

### 8.4. Antitumor Properties

**Figure 53:**
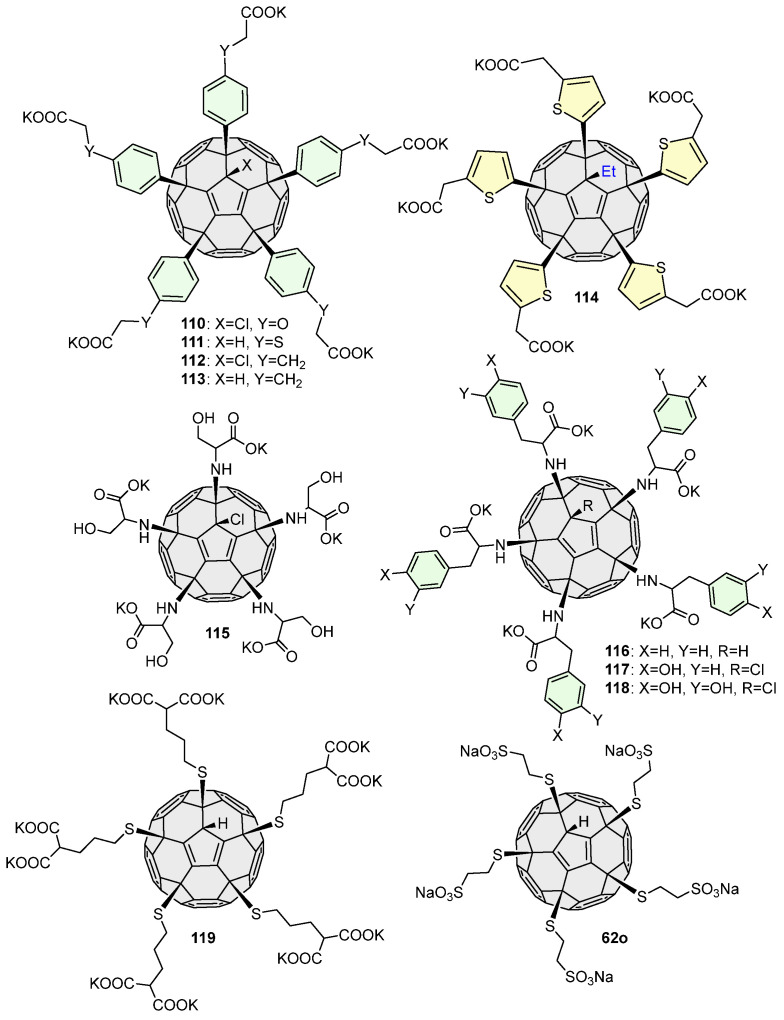
Water-soluble fullerene derivatives demonstrating promising anitumor properties.

Some water-soluble fullerene derivatives exhibit promising antitumor activity through selective mechanisms that spare normal cells. For example, amino acid- and thioacid-functionalized derivatives **110**–**119** and **62o** effectively inhibit lung cancer cell proliferation by inducing either autophagy or apoptosis (Figure 53) [86,158,159,160]. Structural analysis through QSAR modeling reveals that specific molecular features, particularly thiophene moieties, enhance their inhibitory effects against various non-small cell lung carcinoma cell lines [158]. Importantly, these compounds show low acute toxicity in mice, distinguishing them from conventional chemotherapeutics. Some derivatives demonstrate the ability to cross the blood–brain barrier, offering potential dual functionality—either promoting neural regeneration or suppressing glioblastoma growth, depending on their specific functional groups [159]. Their anticancer activity appears to be closely linked to molecular structure, with aromatic bonds, sulfur-containing aromatic rings, and oxygen atoms promoting the inhibitory effects on H460 and H1299 carcinoma cell lines [160]. These findings highlight the potential of fullerene derivatives as a versatile platform for developing targeted, low-toxicity anticancer therapies with possible applications in both peripheral and central nervous system diseases. The ability to fine-tune biological effects through the strategic modification of functional groups opens new possibilities for precision cancer treatment.

Fullerenes serve as efficient photosensitizers in photodynamic therapy due to their unique ability to absorb light and generate cytotoxic reactive oxygen species (ROS), which selectively destroy cancer cells or pathogenic microorganisms. A number of [light-harvesting antenna]–fullerene structures have already been studied for their application in PDT [161,162], including covalently linked BODIPY−C_60_ [163], methylpheophorbide−C_60_ [164], and chlorin e6−C_60_ dyads [165]. Fullerenes also show great promise as antitumor drug carriers due to their unique cage-like structure, which allows for the efficient encapsulation and delivery of chemotherapeutic agents [166].

### 8.5. Neuroprotective Properties

Fullerene derivatives, including several individual compounds, act as cell protectors under induced neurotoxicity conditions [167,168,169].

Additionally, fullerene derivatives were shown to mitigate ischemia–reperfusion injury by scavenging ROS and reactive nitrogen species (RNS) that are overproduced during oxygen deprivation [170,171].

**Figure 54:**
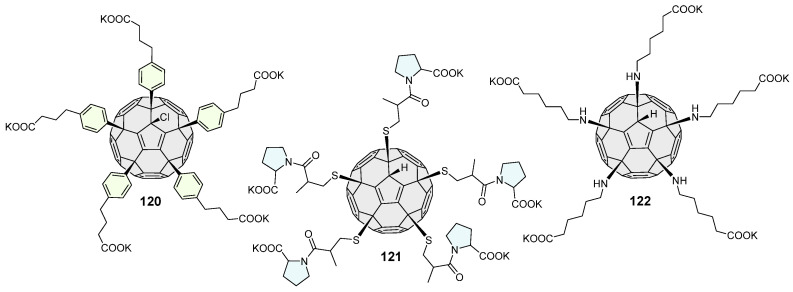
Fullerene derivatives as DNA vectors.

PEGylated, hydroxylated fullerenes and individual polycarboxylic compounds **120**–**122** (Figure 54) inhibit β-amyloid aggregation and destabilize preformed fibrils in vitro and in transgenic Alzheimer’s mouse models [172,173]. Similar effects were observed with α-synuclein [174].

Fullerene derivatives **119**–**121** were observed to induce neural stem cell (NSC) proliferation in vitro and rescue the function of injured CNS in zebrafish. Individual water-soluble fullerenes with different functional groups act on mitochondria that can either induce or reduce the oxidation to achieve neuroprotective or antitumor effects in different types of cells [159].

### 8.6. Fullerene Derivatives as Radioprotectors

**Figure 55:**
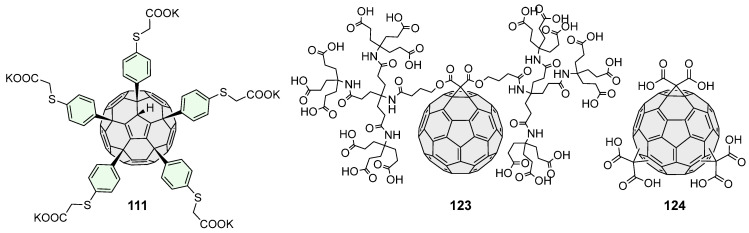
Molecular structures of individual water-soluble fullerene derivatives investigated as potential radioprotectors.

The remarkable antioxidant properties of fullerenes and their derivatives positioned them as promising radioprotective candidates in the early 2000s, when sufficient quantities became available for biological investigation [175]. Initial studies examined both pristine fullerenes and polyhydroxylated mixtures C_60_(OH)_x_ and C_70_(OH)_x_, along with several individual compounds **111**, **123**–**124** (Figure 55). The review paper by Krokosz et al. summarizes most of the data on the radioprotective activity of fullerene derivatives until 2016 [176].

More recent research discusses the influence of non-modified hydrated fullerene [177], and individual compound **124** [178], on cell cultures and animals treated with high-energy electrons, X-rays, and γ-rays. Kostuyk et al. demonstrated that polycarboxylic compound **111** has a stabilizing effect on cell nuclei under the action of 1 Gy radiation [179].

The comprehensive evaluation of these nanomaterials, conducted using in vitro and in vivo animal models, has demonstrated significant radioprotective activity, manifesting as enhanced survival rates in irradiated biological systems, attenuated oxidative stress markers, reduced amounts of radiation-induced DNA damage, and lower intracellular ROS levels.

### 8.7. Fullerene Derivatives as DNA and RNA Vectors

**Figure 56:**
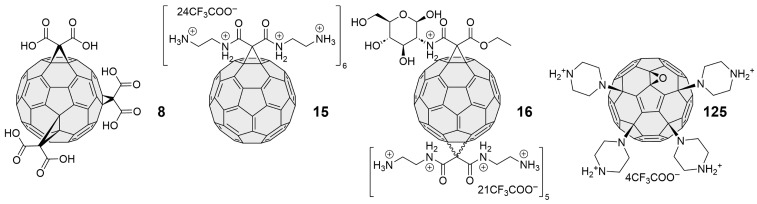
Fullerene derivatives as DNA/RNA vectors.

DNA transfection is the process of intentionally introducing exogenous DNA into the interior of a living cell. Compound **8** (*trans*-3-C_60_A_3_) was utilized as a DNA vector and was able to transfect cells grown in culture with DNA carrying the green fluorescent protein (Figure 56) [53].

siRNA transfection is a molecular biology technique used to deliberately introduce small interfering RNA (siRNA) molecules into eukaryotic cells. siRNA transfection techniques have been actively developed for the tetra-piperazino-[60]fullerene epoxide **125** derivative [180]. Importantly, **125** is non-toxic and achieves lung-targeted in vivo siRNA delivery by forming micrometer-sized complexes with siRNA and serum proteins. These complexes stabilize siRNA, protecting it from degradation under physiological conditions [181]. Recently Wang et al. designed a fullerene-based nanocomplex based on **125** to achieve the pulmonary delivery of siRNA via inhalation. Through the encapsulation of programmed death-ligand 1 (PD-L1) siRNA, this nanocomplex significantly inhibited the progression of metastatic lung cancer in a mouse model, without causing obvious adverse events or toxicity [182]. Polycationic fullerene hexakis adducts **15** and **16** were tested as small interfering RNA (siRNA) vehicles in vitro using the prostate cancer cell line DU145 expressing green fluorescent protein. Compound **15** was an efficient siRNA transfection agent, while glycofullerene **16** was inactive in the transfection experiments due to its high zeta potential and the formation of an extremely stable complex with siRNA [56].

### 8.8. Bactericidal Effects of Fullerene Derivatives

**Figure 57:**
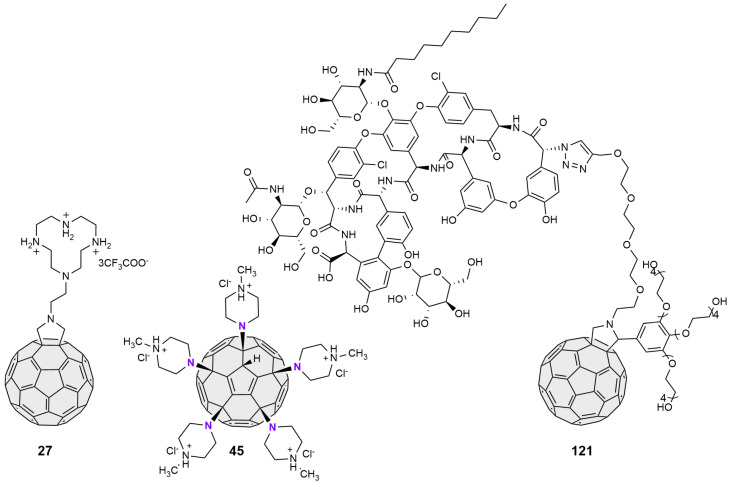
Individual fullerene derivatives possessing antibacterial properties.

In addition to their antiviral properties, certain fullerene monoderivatives have demonstrated significant antibacterial activity (Figure 57). For example, the fullerene–cyclen derivative **27** [183], exhibited potent antibacterial effects against *E. coli* and *S. aureus*. Furthermore, a fullerene–glycopeptide conjugate derived from teicoplanin **126**, showed enhanced antibacterial activity against teicoplanin-resistant *E. faecalis* [184]. In vitro studies demonstrated that polycationic fullerene derivatives, including compound **45**, exhibit potent bactericidal effects against both Gram-negative (*E. coli*) and Gram-positive (*B. subtilis*) bacterial strains. This activity arises from the compound’s ability to assemble on negatively charged cell walls through electrostatic interactions, ultimately disrupting cellular integrity [82].

### 8.9. Fullerene Derivatives in Biomedical Imaging

The accumulation of fullerenes in cells is particularly difficult to measure using standard fluorescence microscopy because their fluorescence is barely detectable in aqueous media [185]. By making an otherwise-invisible nanoparticle visible, fluorescent fullerenes allow researchers to track both the location and activity of these particles simultaneously.

In contrast to pristine fullerenes, multi-functionalized fullerene derivatives exhibit remarkable fluorescent properties due to the partially reduced π-conjugation of the fullerene cage. One approach to achieving this involves the direct chemical modification of the fullerene cage through multi-functionalization. For instance, Rubin, Wudl, and colleagues reported a C_60_ hexaadduct that exhibited significantly improved fluorescence compared to unmodified C_60_ [186]. Similarly, Nakamura and co-workers prepared various C_60_ multiadducts that showed greatly enhanced luminescence relative to pristine C_60_, achieving a maximum quantum yield of 0.24 [187]. Gan et al. have reported that the emission wavelength of C_70_ derivatives obtained via the functionalization of C_70_(OMe)_8_ can be fine-tuned over a wide range from blue to red [188]. Deng et al. have demonstrated that isomerically pure fullerene derivatives C_70_(OCH_3_)_10_[C(COOEt)_2_] and C_70_(OCH_3_)_10_[C(COOEt)_2_]_2_ exhibited enhanced blue fluorescence emission compared to C_70_(OCH_3_)_10_ [189]. The second approach to obtaining fluorescent fullerene derivatives is their conjugation with separate fluorescent molecules. For instance, a fullerene-based photosensitizer has been successfully synthesized by introducing a fullerene unit onto rhodamine B hydrazide [190]. Under acidic conditions, the resulting covalent conjugate, which initially adopts a non-fluorescent spirolactam structure, can be activated to its ring-opened form. This activation accomplishes enhanced visible-light absorbance, fluorescence turn-on, and triplet excited state generation. The fluorescence turn-on and efficient singlet oxygen generation enabled by pH-activated fullerene-based particles testify to their great potential applications for cancer diagnosis and treatment.

T. Cai et al. have developed tumor-targeted C_60_ conjugates, which can be detectable using positron emission tomography (PET) imaging. Fullerene malonic acid tris-adduct was covalently conjugated with eight-arm amine-terminal branched polyethylene glycol. Water-soluble functionalized C_60_ conjugates were radio-labeled with ^64^Cu and modified with cyclo (Arg-GlyAsp) peptides (cRGD) to target integrin α_v_β_3_ in glioblastoma. Although the obtained material was not an individual compound, this approach demonstrates the usability of individual tris-adducts for PET imaging [191]. Virta et al. have created ^18^F-radiolabelled molecular spherical nucleic acids, assembled on a [60]fullerene core via the Bingel–Hirsch reaction and click chemistry. The biodistribution of the compound was monitored in vivo using positron emission tomography/computed tomography (PET/CT) imaging [192].

Gadofullerenes, a class of gadolinium-based metallofullerenes, represent a promising new type of MRI contrast agents with high relaxivities [193]. However, only a few individual water-soluble gadofullerenes were synthesized and investigated. For example, water-soluble Gd metallobuckytrios **22a**–**f** exhibited superior *T*_1_ relaxivity compared to the typical Gd complexes, potentially superseding current clinical MRI contrast agents in both safety and efficiency. The Lu MBTs generated reactive oxygen species upon light irradiation, showing promise as photosensitizers [63].

Other examples are mostly represented by polyhydroxylated, carboxylated, and PEGylated–hydroxylated complex mixtures of gadolinium-based metallofullerenes [194].

### 8.10. Myogenic Activity

Phosphonate C_60_ fullerene derivatives, especially compound **64**, enhanced myogenic differentiation in human mesenchymal stem cells (MSCs) by upregulating key myogenic transcription factors (MYOD1, MYOG, MYF5, and MRF4) while suppressing osteogenic and adipogenic gene expression. The observed myogenic induction may be linked to autophagy stimulation, potentially triggered by intracellular alkalosis or transient oxidative stress caused by the fullerene derivatives. These findings suggest that water-soluble C_60_ derivatives could be promising candidates for regenerative therapies targeting muscle disorders and injuries directing the myogenic differentiation of stem cells [96,195].

### 8.11. Other Types of Biological Activity

Compound **21** exhibits a reactive SpyTag fragment on the fullerene cage utilized for its facile conjugation with SpyCatcher-bearing proteins through the formation of an intermolecular isopeptide bond. The water solubility of this compound was ensured by the addition of multiple residues bearing hydroxyl groups. The efficient, cell-compatible SpyTag–SpyCatcher reaction enabled the native conjugation between fullerenes and protein molecules [62].

The water-soluble glycofullerenes **13** and **14** containing D-glucosamine fragments were proposed as a novel class of non-receptor tyrosine kinase inhibitors, particularly found to inhibit Fyn A tyrosine kinase and Bruton’s tyrosine kinase with IC_50_ values in the low micromolar range [55]. Several individual polycarboxylic C_60_ and C_70_ fullerene derivatives were proposed as protein tyrosine phosphatase inhibitors [196].

## 9. Conclusions and Outlook

Fullerene C_60_ and its functional derivatives exhibit unique properties such as high electron affinity, radical scavenging ability, photodynamic activity, ability to inhibit a wide range of pathogens, etc. However, their poor solubility in water limits their applications in the biomedical field. The development of efficient, tunable synthetic methodologies for water-soluble fullerenes is essential for bridging the gap between their remarkable theoretically predicted properties and real-world applications.

In this review, we have highlighted some general strategies and approaches to the synthesis of individual water-soluble fullerene-based compounds. Namely, classic approaches, such as Bingel–Hirsch and Prato reactions, were discussed; the regioselective multiaddition of organocopper reagents was also introduced as an efficient route to polyfunctional fullerenes. The role of chlorofullerenes as precursors for the synthesis of water-soluble fullerene derivatives was discussed. Importantly, recently developed methodologies for the regioselective synthesis of fullerene derivatives with several addend types were analyzed.

Moving forward, challenges for the biomedical applications of fullerenes remain in their regioselective synthesis and its scalability. The continued development of structurally defined fullerene derivatives holds great promise for drug discovery, nanomedicine, and beyond. Future research should focus on optimizing these materials for practical applications while deepening our understanding of their biological properties and interactions with biological matter.

## Data Availability

No new data were created or analyzed in this study. Data sharing is not applicable to this article.

## References

[1] Kroto H.W., Heath J.R., O’Brien S.C., Curl R.F., Smalley R.E. (1985). C_60_: Buckminsterfullerene. Nature.

[2] Krätschmer W., Lamb L.D., Fostiropoulos K., Huffman D.R. (1990). Solid C_60_: A new form of carbon. Nature.

[3] Diederich F., Thilgen C. (1996). Covalent Fullerene Chemistry. Science.

[4] Bingel C. (1993). Cyclopropanierung von Fullerenen. Chem. Ber..

[5] Hirsch A., Lamparth I., Karfunkel H.R. (1994). Fullerene Chemistry in Three Dimensions: Isolation of Seven Regioisomeric Bisadducts and Chiral Trisadducts of C_60_ and Di(ethoxycarbonyl)methylene. Angew. Chem. Int. Ed..

[6] Chen M., Zeng Y., Chen G., Qiu Y. (2022). Highly Regioselective Synthesis of Bisadduct[C_70_] Additive toward the Enhanced Performance of Perovskite Solar Cells. Nanomaterials.

[7] Bingel C., Schiffer H. (1995). Biscyclopropanation of C_70_. Liebigs Ann..

[8] Maggini M., Scorrano G., Prato M. (1993). Addition of azomethine ylides to C_60_: Synthesis, characterization, and functionalization of fullerene pyrrolidines. J. Am. Chem. Soc..

[9] Birkett P.R., Avent A.G., Darwish A.D., Hahn I., Kroto H.W., Langley G.J., O’Loughlin J., Taylor R., Walton D.R.M. (1997). Arylation of [60]fullerene via electrophilic aromatic substitution involving the electrophile C_60_Cl_6_: Frontside nucleophilic substitution of fullerenes. J. Chem. Soc. Perkin Trans. 2..

[10] Avent A.G., Birkett P.R., Crane J.D., Darwish A.D., Langley G.J., Kroto H.W., Taylor R., Walton D.R.M. (1994). The structure of C_60_Ph_5_Cl and C_60_Ph_5_H, formed via electrophilic aromatic substitution. J. Chem. Soc. Chem. Commun..

[11] Taylor R.C.R. (2006). Addition reactions of fullerenes. Comptes Rendus Chim..

[12] Avent A.G., Birkett P.R., Darwish A.D., Houlton S., Taylor R., Thomson K.S.T., Wei X.-W. (2001). Formation and characterisation of alkoxy derivatives of [60]fullerene. J. Chem. Soc. Perkin Trans. 2.

[13] Birkett P.R., Avent A.G., Darwish A.D., Kroto H.W., Taylor R., Walton D.R.M. (1993). Preparation and ^13^C NMR spectroscopic characterisation of C_60_Cl_6_. J. Chem. Soc. Chem. Commun..

[14] Birkett P.R., Avent A.G., Darwish A.D., Kroto H.W., Taylor R., Walton D.R.M. (1995). Formation and characterisation of C_70_Cl_10_. J. Chem. Soc. Chem. Commun..

[15] Xiao Z., Wang F., Huang S., Gan L., Zhou J., Yuan G., Lu M., Pan J. (2005). Regiochemistry of [70]Fullerene:  Preparation of C_70_(OOtBu)_n_ (n = 2, 4, 6, 8, 10) through Both Equatorial and Cyclopentadienyl Addition Modes. J. Org. Chem..

[16] Gao R., Liu Z., Liu Z., Liang T., Su J., Gan L. (2023). Open-Cage Fullerene as a Selective Molecular Trap for LiF/[BeF]^+^. Angew. Chem. Int. Ed..

[17] Sun S., Liu Z., Colombo F., Gao R., Yu Y., Qiu Y., Su J., Gan L. (2022). Open-Cage Fullerene as Molecular Container for F^−^, Cl^−^, Br^−^ and I^−^. Angew. Chem. Int. Ed..

[18] Li Y., Lou N., Xu D., Pan C., Lu X., Gan L. (2018). Oxygen-Delivery Materials: Synthesis of an Open-Cage Fullerene Derivative Suitable for Encapsulation of H_2_O_2_ and O_2_. Angew. Chem. Int. Ed..

[19] Xin N., Huang H., Zhang J., Dai Z., Gan L. (2012). Fullerene Doping: Preparation of Azafullerene C_59_NH and Oxafulleroids C_59_O_3_ and C_60_O_4_. Angew. Chem. Int. Ed..

[20] Sawamura M., Iikura H., Nakamura E. (1996). The First Pentahaptofullerene Metal Complexes. J. Am. Chem. Soc..

[21] Nakamura E., Sawamura M. (2001). Chemistry of η5-fullerene metal complexes. Pure Appl. Chem..

[22] Yang S., Ioffe I.N., Troyanov S.I. (2019). Chlorination-Promoted Skeletal Transformations of Fullerenes. Acc. Chem. Res..

[23] Wang S., Chang Q., Zhang G., Li F., Wang X., Yang S., Troyanov S.I. (2020). Structural Studies of Giant Empty and Endohedral Fullerenes. Front. Chem..

[24] Troyanov S.I., Kemnitz E. (2005). Synthesis and Structures of Fullerene Bromides and Chlorides. Eur. J. Org. Chem..

[25] Ignat’eva D.V., Ioffe I.N., Troyanov S.I., Sidorov L.N. (2011). Exohedral and skeletal rearrangements in the molecules of fullerene derivatives. Russ. Chem. Rev..

[26] Rašović I. (2017). Water-soluble fullerenes for medical applications. Mater. Sci. Technol..

[27] Poletaeva D.A., Kotelnikova R.A., Faingold I.I., Kraevaya O.A., Troshin P.A., Kotelnikov A.I. (2023). Electrostatic effects on water-soluble fullerene derivatives interaction with cytochrome c and cytochrome c oxidase. J. Biol. Phys..

[28] Gilmutdinova A.A., Gubskaya V.P., Fazleeva G.M., Latypov S.K., Zhelonkina T.A., Sharafutdinova D.R., Nuretdinov I.A., Sinyashin O.G. (2014). Synthesis and properties of new fullerene C_60_ derivatives, containing acetonide and polyol fragments. Tetrahedron.

[29] Shuster D.I., Wilson S.R., Kirschner A.N., Schinazi R.F., Schlueter-Wirtz S., Tharnish P., Barnett T., Ermolieff J., Tang J., Brettreich M. (2000). Evaluation of the anti-HIV potency of a water-soluble dendrimeric fullerene. Proc. Electrochem. Soc..

[30] Brettreich M., Hirsch A. (1998). A highly water-soluble dendro[60]fullerene. Tetrahedron Lett..

[31] Cerón M.R., Echegoyen L.J. (2016). Recent progress in the synthesis of regio-isomerically pure bis-adducts of empty and endohedral fullerenes. Phys. Org. Chem..

[32] Isaacs L., Haldimann R.F., Diederich F. (1994). Tether-Directed Remote Functionalization of Buckminsterfullerene: Regiospecific Hexaadduct Formation. Angew. Chem. Int. Ed..

[33] Ðorđević L., Casimiro L., Demitri N., Baroncini M., Silvi S., Arcudi F., Credi A., Prato M. (2021). Light-Controlled Regioselective Synthesis of Fullerene Bis-Adducts. Angew. Chem. Int. Ed..

[34] Kräutler B., Müller T., Maynollo J., Gruber K., Kratky C., Ochsenbein P., Schwarzenbach D., Bürgi H.-B. (1996). A Topochemically Controlled, Regiospecific Fullerene Bisfunctionalization. Angew. Chem. Int. Ed..

[35] Neti V.S.P.K., Cerón M.R., Duarte-Ruiz A., Olmstead M.M., Balch A.L., Echegoyen L. (2014). High-yield, regiospecific bis-functionalization of C_70_ using a Diels–Alder reaction in molten anthracene. Chem. Commun..

[36] Khakina E.A., Troshin P.A. (2017). Halogenated fullerenes as precursors for the synthesis of functional derivatives of C_60_ and C_70_. Russ. Chem. Rev..

[37] Kraevaya O.A., Chernyak A.V., Shestakov A.F., Troshin P.A. (2024). Displacement of methoxy groups by thiol residues on the fullerene cage. Mendeleev Commun..

[38] Iannace V., Sabrià C., Xu Y., von Delius M., Imaz I., Maspoch D., Feixas F., Ribas X. (2024). Regioswitchable Bingel Bis-Functionalization of Fullerene C_70_ via Supramolecular Masks. J. Am. Chem. Soc..

[39] Chang X., Xu Y., von Delius M. (2024). Recent advances in supramolecular fullerene chemistry. Chem. Soc. Rev..

[40] Hasegawa S., Clever G.H. (2020). Metallo-supramolecular Shell Enables Regioselective Multi-functionalization of Fullerenes. Chem.

[41] Iwamoto T., Watanabe Y., Sadahiro T., Haino T., Yamago S. (2011). Size-Selective Encapsulation of C_60_ by [10]Cycloparaphenylene: Formation of the Shortest Fullerene-Peapod. Angew. Chem. Int. Ed..

[42] Xu Y., Kaur R., Wang B., Minameyer M.B., Gsänger S., Meyer B., Drewello T., Guldi D.M., von Delius M. (2018). Concave–Convex π–π Template Approach Enables the Synthesis of [10]Cycloparaphenylene–Fullerene [2]Rotaxanes. J. Am. Chem. Soc..

[43] García-Simón C., Garcia-Borràs M., Gómez L., Parella T., Osuna S., Juanhuix J., Imaz I., Maspoch D., Costas M., Ribas X. (2014). Sponge-like molecular cage for purification of fullerenes. Nat. Commun..

[44] Castanyer C., Pèlachs T., Romero-Cavagnaro E.A., Sabrià C., Feixas F., Ribas X., Roglans A., Pla-Quintana A. (2026). Supramolecular masks for the regioselective synthesis of Diels–Alder hetero-tris-adduct C_60_ fullerene. Org. Chem. Front..

[45] Ubasart E., Borodin O., Fuertes-Espinosa C., Xu Y., García-Simón C., Gómez L., Juanhuix J., Gándara F., Imaz I., Maspoch D. (2021). A three-shell supramolecular complex enables the symmetry-mismatched chemo- and regioselective bis-functionalization of C_60_. Nat. Chem..

[46] Thilgen C., Wennemers H., Carell T. (2021). François Diederich (1952–2020): 40 Years of Organic Chemistry. Angew. Chem. Int. Ed..

[47] Wang M., Huang L., Sharma S.K., Jeon S., Thota S., Sperandio F.F., Nayka S., Chang J., Hamblin M.R., Chiang L.Y. (2012). Synthesis and Photodynamic Effect of New Highly Photostable Decacationically Armed [60]- and [70]Fullerene Decaiodide Monoadducts To Target Pathogenic Bacteria and Cancer Cells. J. Med. Chem..

[48] Chu C.-C., Ho T.-I., Wang L. (2006). Synthesis and Characterization of C_60_-Anchored Multiarmed Polymers with Well-Defined Structures. Macromolecules.

[49] Xiao S.-L., Wang Q., Yu F., Peng Y.-Y., Yang M., Sollogoub M., Sinaÿ P., Zhang Y.-M., Zhang L.-H., Zhou D.-M. (2012). Conjugation of cyclodextrin with fullerene as a new class of HCV entry inhibitors. Bioorg. Med. Chem..

[50] Zhu X., Xiao S., Zhou D., Sollogoub M., Zhang Y. (2018). Design, synthesis and biological evaluation of water-soluble per-O-methylated cyclodextrin-C_60_ conjugates as anti-influenza virus agents. Eur. J. Med. Chem..

[51] Lamparth I., Hirsch A. (1994). Water-soluble malonic acid derivatives of C_60_ with a defined three-dimensional structure. J. Chem. Soc. Chem. Commun..

[52] Cataldo F., Da Ros T. (2008). Medicinal Chemistry and Pharmacological Potential of Fullerenes and Carbon Nanotubes; Chapter 3.

[53] Sitharaman B., Zakharian T.Y., Saraf A., Misra P., Ashcroft J., Pan S., Pham Q.P., Mikos A.G., Wilson L.J., Engler D.A. (2008). Water-Soluble Fullerene (C_60_) Derivatives as Nonviral Gene-Delivery Vectors. Mol. Pharm..

[54] Mirakyan A.L., Wilson L.J. (2002). Functionalization of C_60_ with diphosphonate groups: A route to bone-vectored fullerenes. J. Chem. Soc. Perkin Trans..

[55] Serda M., Malarz K., Mrozek-Wilczkiewicz A., Wojtyniak M., Musioł R., Curley S.A. (2020). Glycofullerenes as non-receptor tyrosine kinase inhibitors- towards better nanotherapeutics for pancreatic cancer treatment. Sci. Rep..

[56] Korzuch J., Rak M., Balin K., Zubko M., Głowacka O., Dulski M., Musioł R., Madeja Z., Serda M. (2021). Towards water-soluble [60]fullerenes for the delivery of siRNA in a prostate cancer model. Sci. Rep..

[57] Wharton T., Wilson L.J. (2002). Highly-Iodinated Fullerene as a Contrast Agent For X-ray Imaging. Bioorg. Med. Chem..

[58] Nierengarten J.-F., Iehl J., Oerthel V., Holler M., Illescas B.M., Muñoz A., Martín N., Rojo J., Sánchez-Navarro M., Cecioni S. (2010). Fullerene sugar balls. Chem. Commun..

[59] Luczkowiak J., Muñoz A., Sánchez-Navarro M., Ribeiro-Viana R., Ginieis A., Illescas B.M., Martín N., Delgado R., Rojo J. (2013). Glycofullerenes Inhibit Viral Infection. Biomacromolecules.

[60] Muñoz A., Sigwalt D., Illescas B.M., Luczkowiak J., Rodríguez-Pérez L., Nierengarten I., Holler M., Remy J.-S., Buffet K., Vincent S.P. (2016). Synthesis of giant globular multivalent glycofullerenes as potent inhibitors in a model of Ebola virus infection. Nat. Chem..

[61] Ramos-Soriano J., Reina J.J., Illescas B.M., de la Cruz N., Rodríguez-Pérez L., Lasala F., Rojo J., Delgado R., Martín N. (2019). Synthesis of Highly Efficient Multivalent Disaccharide/[60]Fullerene Nanoballs for Emergent Viruses. J. Am. Chem. Soc..

[62] Yin G., Wei J., Shao Y., Wu W.-H., Xu L., Zhang W.-B. (2021). Native conjugation between proteins and [60]fullerene derivatives using SpyTag as a reactive handle. Chin. Chem. Lett..

[63] Li Y., Biswas R., Kopcha W.P., Dubroca T., Abella L., Sun Y., Crichton R.A., Rathnam C., Yang L., Yeh Y.-W. (2023). Structurally Defined Water-Soluble Metallofullerene Derivatives towards Biomedical Applications. Angew. Chem. Int. Ed..

[64] Troshin P.A., Lyubovskaya R.N. (2008). Organic chemistry of fullerenes: The major reactions, types of fullerene derivatives and prospects for their practical use. Russ. Chem. Rev..

[65] Prato M., Maggini M., Giacometti C., Scorrano G., Sandonà G., Farnia G. (1996). Synthesis and electrochemical properties of substituted fulleropyrrolidines. Tetrahedron.

[66] Lu Q., Schuster D.I., Wilson S.R. (1996). Preparation and Characterization of Six Bis(N-methylpyrrolidine)−C_60_ Isomers:  Magnetic Deshielding in Isomeric Bisadducts of C_60_. J. Org. Chem..

[67] Chhabra N., Aseri M.L., Padmanabhan D. (2013). A review of drug isomerism and its significance. Int. J. Appl. Basic Med. Res..

[68] Da Ros T., Prato M., Novello F., Maggini M., Banfi E. (1996). Easy Access to Water-Soluble Fullerene Derivatives via 1,3-Dipolar Cycloadditions of Azomethine Ylides to C_60_. J. Org. Chem..

[69] Aroua S., Schweizer W.B., Yamakoshi Y. (2014). C_60_ Pyrrolidine Bis-carboxylic Acid Derivative as a Versatile Precursor for Biocompatible Fullerenes. Org. Lett..

[70] Biswas R., Da Rocha C.B., Bennick R.A., Zhang J. (2023). Water-Soluble Fullerene Monoderivatives for Biomedical Applications. ChemMedChem.

[71] Marchesan S., Da Ros T., Spalluto G., Balzarini J., Prato M. (2005). Anti-HIV properties of cationic fullerene derivatives. Bioorg. Med. Chem. Lett..

[72] Bosi S., Da Ros T., Spalluto G., Balzarini J., Prato M. (2003). Synthesis and anti-HIV properties of new water-soluble bis-functionalized[60]fullerene derivatives. Bioorg. Med. Chem. Lett..

[73] Matsuo Y., Nakamura E. (2008). Selective Multiaddition of Organocopper Reagents to Fullerenes. Chem. Rev..

[74] Matsuo Y., Ichiki T., Radhakrishnan S.G., Guldi D.M., Nakamura E. (2010). Loading Pentapod Deca(organo)[60]fullerenes with Electron Donors: From Photophysics to Photoelectrochemical Bilayers. J. Am. Chem. Soc..

[75] Zhou S., Burger C., Chu B., Sawamura M., Nagahama N., Toganoh M., Hackler U.E., Isobe H., Nakamura E. (2001). Spherical Bilayer Vesicles of Fullerene-Based Surfactants in Water: A Laser Light Scattering Study. Science.

[76] Isobe H., Cho K., Solin N., Werz D.B., Seeberger P.H., Nakamura E. (2007). Synthesis of Fullerene Glycoconjugates via a Copper-Catalyzed Huisgen Cycloaddition Reaction. Org. Lett..

[77] Nitta H., Minami K., Harano K., Nakamura E. (2015). DNA Binding of Pentaamino[60]fullerene Synthesized Using Click Chemistry. Chem. Lett..

[78] Tebbe F.N., Becker J.Y., Chase D.B., Firment L.E., Holler E.R., Malone B.S., Krusic P.J., Wasserman E. (1991). Multiple, reversible chlorination of C_60_. J. Am. Chem. Soc..

[79] Olah G.A., Bucsi I., Lambert C., Aniszfeld R., Trivedi N.J., Sensharma D.K., Prakash G.K.S. (1991). Chlorination and bromination of fullerenes. Nucleophilic methoxylation of polychlorofullerenes and their aluminum trichloride catalyzed Friedel-Crafts reaction with aromatics to polyarylfullerenes. J. Am. Chem. Soc..

[80] Kuvychko I.V., Streletskii A.V., Popov A.A., Kotsiris S.G., Drewello T., Strauss S.H., Boltalina O.V. (2005). Seven-Minute Synthesis of Pure C_s_-C_60_Cl_6_ from [60]Fullerene and Iodine Monochloride: First IR, Raman, and Mass Spectra of 99 mol % C_60_Cl_6_. Chem. Eur. J..

[81] Troshin P.A., Popkov O., Lyubovskaya R.N. (2003). Some New Aspects of Chlorination of Fullerenes. Fuller. Nanotub. Carbon Nanostruct..

[82] Kornev A.B., Khakina E.A., Troyanov S.I., Kushch A.A., Peregudov A., Vasilchenko A., Deryabin D.G., Martynenko V.M., Troshin P.A. (2012). Facile preparation of amine and amino acid adducts of [60]fullerene using chlorofullerene C_60_Cl_6_ as a precursor. Chem. Commun..

[83] Hu Z., Zhang C., Huang Y., Sun S., Guan W., Yao Y. (2012). Photodynamic anticancer activities of water-soluble C(60) derivatives and their biological consequences in a HeLa cell line. Chem. Biol. Interact..

[84] Romanova V.S., Tsyryapkin V.A., Lyakhovetsky Y.I., Parnes Z.N., Vol’pin M.E. (1994). Addition of amino acids and dipeptides to fullerene C_60_ giving rise to monoadducts. Russ. Chem. Bull..

[85] Hu Z., Huang Y., Guan W., Zhang J., Wang F., Zhao L. (2010). The protective activities of water-soluble C_60_ derivatives against nitric oxide-induced cytotoxicity in rat pheochromocytoma cells. Biomaterials.

[86] Wong C.-W., Zhilenkov A.V., Kraevaya O.A., Mischenko D.V., Troshin P.A., Hsu S.-H. (2019). Toward Understanding the Antitumor Effects of Water-Soluble Fullerene Derivatives on Lung Cancer Cells: Apoptosis or Autophagy Pathways?. J. Med. Chem..

[87] Lin M.-S., Chen R.-T., Yu N.-Y., Sun L.-C., Liu Y., Cui C.-H., Xie S.-Y., Huang R.-B., Zheng L.-S. (2017). Fullerene-based amino acid ester chlorides self-assembled as spherical nano-vesicles for drug delayed release. Colloids Surf. B Biointerfaces.

[88] Deng L.-L., Xie S.-L., Yuan C., Liu R.-F., Feng J., Sun L.-C., Lu X., Xie S.-Y., Huang R.-B., Zheng L.-S. (2013). High LUMO energy level C_60_(OCH_3_)_4_ derivatives: Electronic acceptors for photovoltaic cells with higher open-circuit voltage. Sol. Energy Mater. Sol. Cells.

[89] Khakina E.A., Kraevaya O.A., Popova M.L., Peregudov A.S., Troyanov S.I., Chernyak A.V., Martynenko V.M., Kulikov A.V., Schols D., Troshin P.A. (2017). Synthesis of different types of alkoxy fullerene derivatives from chlorofullerene C_60_Cl_6_. Org. Biomol. Chem..

[90] Kraevaya O.A., Peregudov A.S., Martynenko V.M., Troshin P.A. (2018). Facile synthesis of isomerically pure fullerenols C_60_(OH)_5_Br and 1,4-C_60_(OH)_2_ from chlorofullerene C_60_Cl_6_. Tetrahedron Lett..

[91] Lou N., Gan L. (2017). Synthesis and reactivity of tetraalkoxyl[60]fullerene epoxides, C_60_(O)(OR)_4_. Can. J. Chem..

[92] Khakina E.A., Yurkova A.A., Peregudov A.S., Troyanov S.I., Trush V.V., Vovk A.I., Mumyatov A.V., Martynenko V.M., Balzarini J., Troshin P.A. (2012). Highly selective reactions of C_60_Cl_6_ with thiols for the synthesis of functionalized [60]fullerene derivatives. Chem. Commun..

[93] Novikov A.V., Kuznetsova L.I., Dremova N.N., Parfenov A.A., Troshin P.A. (2020). Environment-friendly aqueous processing of [60]fullerene semiconducting films for truly green organic electronics. J. Mater. Chem. C.

[94] Arbusow B.A. (1964). Michaelis-Arbusow- Und Perkow- Reaktionen. Pure Appl. Chem..

[95] Yurkova A.A., Khakina E.A., Troyanov S.I., Chernyak A., Shmygleva L., Peregudov A.S., Martynenko V.M., Dobrovolskiy Y.A., Troshin P.A. (2012). Arbuzov chemistry with chlorofullerene C_60_Cl_6_: A powerful method for selective synthesis of highly functionalized [60]fullerene derivatives. Chem. Commun..

[96] Kraevaya O.A., Novikov A.V., Shestakov A.F., Ershova E.S., Savinova E.A., Kameneva L.V., Veiko N.N., Schols D., Balzarini J., Kostyuk S.V. (2020). Water-soluble fullerene-based nanostructures with promising antiviral and myogenic activity. Chem. Commun..

[97] Kraevaya O.A., Peregudov A.S., Shestakov A.F., Troshin P.A. (2024). Synthesis of Cs-symmetrical C_60_ tetra-adducts via reactions of C_60_Cl_6_ with CH-acids and enol silyl ester. Org. Biomol. Chem..

[98] Al-Matar H., Abdul-Sada A.K., Avent A.G., Taylor R. (2001). Isolation and Characterization of Unsymmetrical C_60_Me_5_O_3_H, a Cage-Opened Bisepoxide Ketone:  Tautomerism Involving a Fullerene Cage Bond. Org. Lett..

[99] Al-Matar H., Abdul-Sada A.K., Avent A.G., Fowler P.W., Hitchcock P.B., Rogers K.M., Taylor R. (2002). Isolation and characterisation of symmetrical C_60_Me_6_, C_60_Me_5_Cl and C_60_Me_5_O_2_OH, together with unsymmetrical C_60_Me_5_O_3_H, C_60_Me_5_OOH, C_60_Me_4_PhO_2_OH, and C_60_Me_12_; fragmentation of methylfullerenols to C_58_. J. Chem. Soc. Perkin Trans. 2.

[100] Al-Matar H., Hitchcock P.B., Avent A.G., Taylor R. (2000). Isolation and characterisation of Cs-symmetry C_60_Me_5_O_2_OH, the first methylated fullerenol; a bis-epoxide with two oxygens in a pentagonal ring. Chem. Commun..

[101] Abdul-Sada A.K., Avent A.G., Birkett P.R., Kroto H.W., Taylor R., Walton D.R.M. (1998). A hexaallyl[60]fullerene, C_60_(CH_2_CHCH_2_)_6_. J. Chem. Soc. Perkin Trans. 1.

[102] Wang S., Yan P., Huang H.-Y., Zhan Z.-P., Xie S.-Y., Huang R.-B., Zheng L.-S. (2012). Construction of Fullerocyclobutene Derivatives through Copper(I)-Mediated Radical Annulation of C_60_Cl_6_ with Aryl Acetylenes. Chem. Asian J..

[103] Troshin P.A., Khakina E.A., Peregudov A.S., Konarev D.V., Soulimenkov I.V., Peregudova S.M., Lyubovskaya R.N. (2010). [C_60_(CN)_5_]^−^: A Remarkably Stable [60]Fullerene Anion. Eur. J. Org. Chem..

[104] Troshina O.A., Troshin P.A., Peregudov A.S., Kozlovskiy V.I., Balzarini J., Lyubovskaya R.N. (2007). Chlorofullerene C_60_Cl_6_: A precursor for straightforward preparation of highly water-soluble polycarboxylic fullerene derivatives active against HIV. Org. Biomol. Chem..

[105] Zhao Y., Jin B., Ding L., Xiao L., Peng R. (2020). Regioselective synthesis of 4,11,15,30-tetraalkoxyphenyl fullereno[1,2:2′,3′]dihydrobenzofurans and potential application as propellant stabilizer. Tetrahedron Lett..

[106] Ding L., Jin B., Guo Z., Zhao Y., Chen J., Peng R. (2019). Regioselective Synthesis and Crystallographic Characterization of Nontethered cis-1 and cis-2 Bis(benzofuro)[60]fullerene Derivatives. Org. Lett..

[107] Voronov I.I., Martynenko V.M., Chernyak A.V., Godovikov I., Peregudov A.S., Balzarini J., Shestakov A.F., Schols D., Troshin P.A. (2020). Synthesis, characterization and anti-HIV activity of polycarboxylic [60]fullerene derivatives obtained in the reaction of C_60_Cl_6_ with a hydroquinone ether. Tetrahedron Lett..

[108] Kraevaya O.A., Peregudov A.S., Godovikov I.A., Shchurik E.V., Martynenko V.M., Shestakov A.F., Balzarini J., Schols D., Troshin P.A. (2020). Direct arylation of C_60_Cl_6_ and C_70_Cl_8_ with carboxylic acids: A synthetic avenue to water-soluble fullerene derivatives with promising antiviral activity. Chem. Commun..

[109] Bolshakova V.S., Sinegubova E.O., Esaulkova Y.L., Peregudov A.S., Khakina E.A., Slesarenko N.A., Shestakov A.F., Zarubaev V.V., Troshin P.A., Kraevaya O.A. (2023). Facile Synthesis of Amino Acid Decorated Water-Soluble Fullerene Derivatives with Anti-influenza Activity. Chin. J. Chem..

[110] Kraevaya O.A., Peregudov A.S., Troyanov S.I., Godovikov I., Fedorova N.E., Klimova R.R., Sergeeva V.A., Kameneva L.V., Ershova E.S., Martynenko V.M. (2019). Diversion of the Arbuzov reaction: Alkylation of C–Cl instead of phosphonic ester formation on the fullerene cage. Org. Biomol. Chem..

[111] Taylor R. (2000). Surprises, Serendipity, and Symmetry in Fullerene Chemistry. Synlett.

[112] Abdul-Sada A.K., Avent A.G., Walton D.R.M., Woodhouse O.B. (1998). Novel formation of a phenylated isoquinolino[3′,4′:1,2][60]fullerene. Chem. Commun..

[113] Lou N., Li Y., Cui C., Liu Y., Gan L. (2016). Preparation of Azafullerene C_59_NR_5_ and Fullerene Derivative C_60_NAr_5_ with a Pyridine Moiety on the Cage Skeleton. Org. Lett..

[114] Lou N., Kraevaya O.A., Troshin P.A., Gan L. (2018). Synthesis of Pentapyrazolyl, Pentapyrrolyl, and Pentaanilino C_60_ Derivatives. Synthesis.

[115] Boltalina O.V., Markov V.Y., Taylor R., Waugh M.P. (1996). Preparation and characterisation of C_60_F_18_. Chem. Commun..

[116] Darwish A.D., Avent A.G., Abdul-Sada A.K., Gol’dt I.V., Hitchcock P.B., Kuvytchko I.V., Taylor R. (2004). Electrophilic Aromatic Substitution by the Fluorofullerene C_60_F_18_. Chem. Eur. J..

[117] Troshin P.A., Khakina E.A., Zhilenkov A.V., Peregudov A.S., Troshina O.A., Kozlovskii V.I., Polyakova N.V., Lyubovskaya R.N. (2010). Synthesis and Spectroscopic Characterization of the First Symmetrically and Nonsymmetrically Substituted Fluorinated Emerald-Green Trannulenes C_60_F_15_R_3_ Soluble in Polar Media and Water. Eur. J. Org. Chem..

[118] Khakina E.A., Troyanov S.I., Peregudov A.S., Soulimenkov I.V., Polyakova N.V., Troshin P.A. (2010). The Remarkable Chemistry of Trannulenes: Green Fluorinated Fullerenes with Unconventional Aromaticity. Chem. Eur. J..

[119] Voronov I.I., Martynenko V.M., Chernyak A.V., Balzarini J., Schols D., Troshin P.A. (2018). Synthesis and Antiviral Activity of Water-Soluble Polycarboxylic Derivatives of [60]Fullerene Loaded with 3,4-Dichlorophenyl Units. Chem. Biodivers..

[120] Hormann F., Donaubauer W., Hampel F., Hirsch A. (2012). Efficient Synthesis of C2v-Symmetrical Pentakisadducts of C_60_ as Versatile Building Blocks for Fullerene Architectures that Involve a Mixed Octahedral Addition Pattern. Chem. Eur. J..

[121] Su Y.-T., Yin Z.-C., Wang G.-W. (2022). Palladium-Catalyzed Three-Component 1,4-Alkoxyarylation Reaction of [60]Fullerene. J. Org. Chem..

[122] Liu Z., Yin Z.-C., Lu W.-Q., Niu C., Chen M., Yang S., Wang G.-W. (2021). Cu(I)-Catalyzed Synthesis of [60]Fullerene-Fused Lactams and Further Electrochemical Functionalization. Org. Lett..

[123] Li C.-Z., Matsuo Y., Nakamura E. (2011). Regioselective synthesis of tetra(aryl)-mono(silylmethyl)[60]fullerenes and derivatization to methanofullerene compound. Tetrahedron.

[124] Chen S., Yang W.-W., Hou H.-L., Li Z.-J., Gao X. (2021). Reactions of [60]Fullerene with Acetone under Basic Condition: Nucleophilic Ring Opening of the [5,6]-Cyclopropane in C_60_ and Formation of the Substituted Methano[60]Fulleroids. J. Org. Chem..

[125] Bolshakova V.S., Kraevaya O.A., Peregudov A.S., Markov V.Y., Kostyuk S.V., Schols D., Shestakov A.F., Troshin P.A. (2023). A regioselective step-by-step C_60_Cl_6_ functionalization approach affords a novel family of C_60_Ar_5_Th′Th′′H fullerene derivatives with promising antiviral properties. Chem. Commun..

[126] Raoof M., Mackeyev Y., Cheney M.A., Wilson L.J., Curley S.A. (2012). Internalization of C_60_ fullerenes into cancer cells with accumulation in the nucleus via the nuclear pore complex. Biomaterials.

[127] Zhang L.W., Yang J., Barron A.R., Monteiro-Riviere N.A. (2009). Endocytic mechanisms and toxicity of a functionalized fullerene in human cells. Toxicol. Lett..

[128] Foley S., Crowley C., Smaihi M., Bonfils C., Erlanger B.F., Seta P., Larroque C. (2002). Cellular localisation of a water-soluble fullerene derivative. Biochem. Biophys. Res. Commun..

[129] Chirico F., Fumelli C., Marconi A., Tinari A., Straface E., Malorni W., Pellicciari R., Pincelli C. (2007). Carboxyfullerenes localize within mitochondria and prevent the UVB-induced intrinsic apoptotic pathway. Exp. Dermatol..

[130] Li W., Chen C., Ye C., Wei T., Zhao Y., Lao F., Chen Z., Meng H., Gao Y., Yuan H. (2008). The translocation of fullerenic nanoparticles into lysosome via the pathway of clathrin-mediated endocytosis. Nanotechnology.

[131] Corbo C., Molinaro R., Parodi A., Toledano Furman N.E., Salvatore F., Tasciotti E. (2016). The impact of nanoparticle protein corona on cytotoxicity, immunotoxicity and target drug delivery. Nanomedicine.

[132] Serda M., Korzuch J., Dreszer D., Krzykawska-Serda M., Musioł R. (2023). Interactions between modified fullerenes and proteins in cancer nanotechnology. Drug Discov. Today.

[133] Wu L., Fu F., Wang W., Wang W., Huang Z., Huang Y., Pan X., Wu C. (2023). Plasma protein corona forming upon fullerene nanocomplex: Impact on both counterparts. Particuology.

[134] Yang L., Wang Z., Wang Z., Jiang W., Lu Y., Li B., Zhang Z., Li J., Yu F., Guo Q. (2026). Uni-Full: An Artificial Intelligence Model for Accurate Prediction of Protein Targets of Amphiphilic Fullerene Derivatives. CCS Chem..

[135] Bolshakova O., Zherebyatieva O., Sarantseva S.V. (2025). Fullerenes in vivo. Toxicity and protective effects. Nanotoxicology.

[136] Kolosnjaj J., Szwarc H., Moussa F. (2007). Bio-Applications of Nanoparticles.

[137] Franskevych D., Palyvoda K., Petukhov D., Prylutska S., Grynyuk I., Schuetze C., Drobot L., Matyshevska O., Ritter U. (2017). Fullerene C_60_ Penetration into Leukemic Cells and Its Photoinduced Cytotoxic Effects. Nanoscale Res. Lett..

[138] Bolshakova V.A., Kraevaya O.A., Troshin P.A. Synthesis of new water-soluble fullerene derivatives with attached amino acid residues and investigation of their antiviral activity against influenza viruses and SARS-CoV-2 coronavirus. Proceedings of the Presented at the Baturin Conference.

[139] Schinazi R.F., Sijbesma R.P., Srdanov G., Hill C.L., Wudl F. (1993). Synthesis and virucidal activity of a water-soluble, configurationally stable, derivatized C_60_ fullerene. Antimicrob. Agents Chemother..

[140] Friedman S.H., DeCamp D.L., Sijbesma R.P., Srdanov G., Wudl F., Kenyon G.L. (1993). Inhibition of the HIV-1 protease by fullerene derivatives: Model building studies and experimental verification. J. Am. Chem. Soc..

[141] Toniolo A., Bianco A., Maggini M., Scorrano G., Prate M., Marastoni M., Tomatis R., Spisani S., Palu G., Blair E.D. (1994). A Bioactive Fullerene Peptide. J. Med. Chem..

[142] Friedman S.H., Ganapathi P.S., Rubin Y., Kenyon G.L. (1998). Optimizing the binding of fullerene inhibitors of the HIV-1 protease through predicted increases in hydrophobic desolvation. J. Med. Chem..

[143] Strom T.A., Durdagi S., Ersoz S.S., Salmas R.E., Supuran C.T., Barron A.R. (2015). Fullerene-based inhibitors of HIV-1 protease. J. Pept. Sci..

[144] Kraevaya O.A., Peregudov A.S., Fedorova N.E., Klimova R.R., Godovikov I.A., Mishchenko D.V., Shestakov A.F., Schols D., Kushch A.A., Troshin P.A. (2020). Thiophene-based water-soluble fullerene derivatives as highly potent antiherpetic pharmaceuticals. Org. Biomol. Chem..

[145] Sinegubova E.O., Kraevaya O.A., Volobueva A.S., Zhilenkov A.V., Shestakov A.F., Baykov S.V., Troshin P.A., Zarubaev V.V. (2023). Water-Soluble Fullerene C_60_ Derivatives Are Effective Inhibitors of Influenza Virus Replication. Microorganisms.

[146] Reina G., Peng S., Jacquemin L., Andrade A.F., Bianco A. (2020). Hard Nanomaterials in Time of Viral Pandemics. ACS Nano.

[147] Riley P.R., Narayan R.J. (2021). Recent advances in carbon nanomaterials for biomedical applications: A review. Curr. Opin. Biomed. Eng..

[148] Serrano-Aroca Á., Takayama K., Tuñón-Molina A., Seyran M., Hassan S.S., Choudhury P.P., Uversky V.N., Lundstrom K., Adadi P., Palù G. (2021). Carbon-Based Nanomaterials: Promising Antiviral Agents to Combat COVID-19 in the Microbial-Resistant Era. ACS Nano.

[149] Hurmach V.V., Platonov M.O., Prylutska S.V., Scharff P., Prylutskyy Y.I., Ritter U. (2021). C_60_ fullerene against SARS-CoV-2 coronavirus: An in silico insight. Sci. Rep..

[150] Skariyachan S., Gopal D., Deshpande D., Joshi A., Uttarkar A., Niranjan V. (2021). Carbon fullerene and nanotube are probable binders to multiple targets of SARS-CoV-2: Insights from computational modeling and molecular dynamic simulation studies. Infect. Genet. Evol..

[151] Saleh N.A. (2023). In-silico study: Docking simulation and molecular dynamics of peptidomimetic fullerene-based derivatives against SARS-CoV-2 Mpro. 3 Biotech.

[152] Lemos R., Makowski K., Almagro L., Tolón B., Rodríguez H., Herranz M.Á., Molero D., Martín N., Suárez M. (2023). Synthesis of [60]Fullerene Hybrids Endowed with Steroids and Monosaccharides: Theoretical Underpinning as Promising anti-SARS-CoV-2 Agents. Eur. J. Org. Chem..

[153] Katin K.P., Kochaev A.I., Kaya S., El-Hajjaji F., Maslov M.M. (2022). Ab Initio Insight into the Interaction of Metal-Decorated Fluorinated Carbon Fullerenes with Anti-COVID Drugs. Int. J. Mol. Sci..

[154] Zhang F., Wang Z., Vijver M.G., Peijnenburg W.J.G.M. (2021). Probing nano-QSAR to assess the interactions between carbon nanoparticles and a SARS-CoV-2 RNA fragment. Ecotoxicol. Environ. Saf..

[155] Petitjean S.J.L., Chen W., Koehler M., Jimmidi R., Yang J., Mohammed D., Juniku B., Stanifer M.L., Boulant S., Vincent S.P. (2022). Multivalent 9-O-Acetylated-sialic acid glycoclusters as potent inhibitors for SARS-CoV-2 infection. Nat. Commun..

[156] Katagishi D., Yasuda D., Takahashi K., Nakamura S., Mashino T., Ohe T. (2023). Fullerene derivatives as inhibitors of the SARS-CoV-2 main protease. Bioorg. Med. Chem. Lett..

[157] Kraevaya O.A., Bolshakova V.S., Slita A.V., Esaulkova I.L., Zhilenkov A.V., Mikhalsky M.G., Sinegubova E.O., Voronov I.I., Peregudov A.S., Shestakov A.F. (2025). Buckyballs to fight pandemic: Water-soluble fullerene derivatives with pendant carboxylic groups emerge as a new family of promising SARS-CoV-2 inhibitors. Bioorg. Chem..

[158] Huang H.-J., Kraevaya O.A., Voronov I.I., Troshin P.A., Hsu S.-h. (2020). Fullerene Derivatives as Lung Cancer Cell Inhibitors: Investigation of Potential Descriptors Using QSAR Approaches. Int. J. Nanomed..

[159] Hsieh F.-Y., Zhilenkov A.V., Voronov I.I., Khakina E.A., Mischenko D.V., Troshin P.A., Hsu S.-H. (2017). Water-Soluble Fullerene Derivatives as Brain Medicine: Surface Chemistry Determines If They Are Neuroprotective and Antitumor. ACS Appl. Mater. Interfaces.

[160] Huang H.-J., Chetyrkina M., Wong C.-W., Kraevaya O.A., Zhilenkov A.V., Voronov I.I., Wang P.-H., Troshin P.A., Hsu S.-h. (2021). Identification of potential descriptors of water-soluble fullerene derivatives responsible for antitumor effects on lung cancer cells via QSAR analysis. Comput. Struct. Biotechnol. J..

[161] Constantin C., Neagu M., Ion R.-M., Gherghiceanu M., Stavaru C. (2010). Fullerene-porphyrin nanostructures in photodynamic therapy. Nanomedicine.

[162] Li Q., Hong L., Li H., Liu C. (2017). Graphene oxide-fullerene C_60_ (GO-C_60_) hybrid for photodynamic and photothermal therapy triggered by near-infrared light. Biosens. Bioelectron..

[163] Agazzi M.L., Durantini J.E., Gsponer N.S., Durantini A.M., Bertolotti S.G., Durantini E.N. (2019). Light-Harvesting Antenna and Proton-Activated Photodynamic Effect of a Novel BODIPY−Fullerene C_60_ Dyad as Potential Antimicrobial Agent. ChemPhysChem.

[164] Kotelnikov A.I., Rybkin A.Y., Khakina E.A., Kornev A.B., Barinov A.V., Goryachev N.S., Ivanchikhina A.V., Peregudov A.S., Martynenko V.M., Troshin P.A. (2013). Hybrid photoactive fullerene derivative–ruboxyl nanostructures for photodynamic therapy. Org. Biomol. Chem..

[165] Rybkin A.Y., Belik A.Y., Tarakanov P.A., Taziev K.R., Kozlov A.V., Goryachev N.S., Sulimenkov I.V., Kozlovskiy V.I., Romanenko Y.V., Koifman O.I. (2019). Pyropheophorbide-Fullerene Dyad: Synthesis and Photochemical Properties. Macroheterocycles.

[166] Kumar M., Raza K. (2017). C_60_-fullerenes as Drug Delivery Carriers for Anticancer Agents: Promises and Hurdles. Pharm. Nanotechnol..

[167] Lin A.M.Y., Chyi B.Y., Wang S.D., Yu H.-H., Kanakamma P.P., Luh T.-Y., Chou C.K., Ho L.T. (1999). Carboxyfullerene Prevents Iron-Induced Oxidative Stress in Rat Brain. J. Neurochem..

[168] Dugan L.L., Turetsky D.M., Du C., Lobner D., Wheeler M., Almli C.R., Shen C.K., Luh T.Y., Choi D.W., Lin T.S. (1997). Carboxyfullerenes as neuroprotective agents. Proc. Natl. Acad. Sci. USA.

[169] Giust D., Da Ros T., Martín M., Albasanz J.L. (2014). [60]Fullerene derivative modulates adenosine and metabotropic glutamate receptors gene expression: A possible protective effect against hypoxia. J. Nanobiotechnol..

[170] Yang D.-Y., Wang M.-F., Chen I.-L., Chan Y.-C., Lee M.-S., Cheng F.-C. (2001). Systemic administration of a water-soluble hexasulfonated C(_60_) (FC(4)S) reduces cerebral ischemia-induced infarct volume in gerbils. Neurosci. Lett..

[171] Darabi S., Mohammadi M.T. (2017). Fullerenol nanoparticles decrease ischaemia-induced brain injury and oedema through inhibition of oxidative damage and aquaporin-1 expression in ischaemic stroke. Brain Inj..

[172] Lee C.-M., Huang S.-T., Huang S.-H., Lin H.-W., Tsai H.-P., Wu J.-Y., Lin C.-M., Chen C.-T. (2011). C_60_ fullerene-pentoxifylline dyad nanoparticles enhance autophagy to avoid cytotoxic effects caused by the β-amyloid peptide. Nanomedicine.

[173] Siposova K., Petrenko V.I., Ivankov O.I., Musatov A., Bulavin L.A., Avdeev M.V., Kyzyma O.A. (2020). Fullerenes as an Effective Amyloid Fibrils Disaggregating Nanomaterial. ACS Appl. Mater. Interfaces.

[174] Bobylev A.G., Kraevaya O.A., Bobyleva L.G., Khakina E.A., Fadeev R.S., Zhilenkov A.V., Mishchenko D.V., Penkov N.V., Teplov I.Y., Yakupova E.I. (2019). Anti-amyloid activities of three different types of water-soluble fullerene derivatives. Colloids Surf. B Biointerfaces.

[175] Lin H.-S., Lin T.-S., Lai R.-S., D’Rosario T., Luh T.-Y. (2001). Fullerenes as a new class of radioprotectors. Int. J. Radiat. Biol..

[176] Krokosz A., Lichota A., Nowak K.E., Grebowski J. (2016). Carbon nanoparticles as possible radioprotectors in biological systems. Radiat. Phys. Chem..

[177] Gudkov S.V., Guryev E.L., Gapeyev A.B., Sharapov M.G., Bunkin N.F., Shkirin A.V., Zabelina T.S., Glinushkin A.P., Sevost’yanov M.A., Belosludtsev K.N. (2019). Unmodified hydrated C_60_ fullerene molecules exhibit antioxidant properties, prevent damage to DNA and proteins induced by reactive oxygen species and protect mice against injuries caused by radiation-induced oxidative stress. Nanomedicine.

[178] Cui J., Yang Y., Cheng Y., Gao F., Liu C., Zhou C., Cheng Y., Li B., Cai J. (2013). Protective effects of carboxyfullerene in irradiated cells and BALB/c mice. Free Radic. Res..

[179] Kostyuk S.V., Malinovskaya E.M., Umriukhin P.E., Proskurnina E.V., Ershova E.S., Kameneva L.V., Savinova E.A., Kostyuk S.E., Voronov I.I., Kraevaya O.A. (2024). Cytoprotective Effects and Intranuclear Localization of Sulfur-Containing Derivative of Buckminsterfullerene. Front. Biosci..

[180] Minami K., Okamoto K., Doi K., Harano K., Noiri E., Nakamura E. (2014). siRNA delivery targeting to the lung via agglutination-induced accumulation and clearance of cationic tetraamino fullerene. Sci. Rep..

[181] Minami K., Okamoto K., Harano K., Noiri E., Nakamura E. (2018). Hierarchical Assembly of siRNA with Tetraamino Fullerene in Physiological Conditions for Efficient Internalization into Cells and Knockdown. ACS Appl. Mater. Interfaces.

[182] Liu S., Sun X., Lu H., Chen D., Li X., Li L., Su S., Zhao Z., Cao X., Liu L. (2023). Fullerene-based nanocomplex assists pulmonary delivery of siRNA for treating metastatic lung cancer. Nano Today.

[183] Chen Q., Ma Z., Liu G., Wei H., Xie X. (2016). Antibacterial activity of cationic cyclen-functionalized fullerene derivatives: Membrane stress. Dig. J. Nanomat. Biostruct..

[184] Tollas S., Bereczki I., Sipos A., Rőth E., Batta G., Daróczi L., Kéki S., Ostorházi E., Rozgonyi F., Herczegh P. (2012). Nano-sized clusters of a teicoplanin ψ-aglycon-fullerene conjugate. Synthesis, antibacterial activity and aggregation studies. Eur. J. Med. Chem..

[185] Andreoni A., Nardo L., Bondani M., Zhao B., Roberts J.E. (2013). Time-resolved fluorescence studies of fullerene derivatives. J. Phys. Chem. B.

[186] Hutchison K., Gao J., Schick G., Rubin Y., Wudl F. (1999). Bucky light bulbs: White light electroluminescence from a fluorescent C_60_ adduct-single layer organic LED. J. Am. Chem. Soc..

[187] Matsuo Y., Tahara K., Morita K., Matsuo K., Nakamura E. (2007). Regioselective Eightfold and Tenfold Additions of a Pyridine-Modified Organocopper Reagent to [60]Fullerene. Angew. Chem. Int. Ed..

[188] Lou N., Li Y., Gan L. (2017). Synthesis of C_70_-Based Fluorophores through Sequential Functionalization to Form Isomerically Pure Multiadducts. Angew. Chem..

[189] Luan K., Wang L., Xie F.-F., Chen B.-W., Chen Z.-C., Deng L.-L., Xie S.-Y., Zheng L.-S. (2022). Synthesis and Fluorescent Properties of Multi-Functionalized C_70_ Derivatives of C_70_(OCH_3_)_10_[C(COOEt)_2_] and C_70_(OCH_3_)_10_[C(COOEt)_2_]_2_. Nanomaterials.

[190] Tang Q., Xiao W., Li J., Chen D., Zhang Y., Shao J., Dong X. (2018). A fullerene-rhodamine B photosensitizer with pH-activated visible-light absorbance/fluorescence/photodynamic therapy. J. Mater. Chem. B.

[191] Peng Y., Yang D., Lu W., Hu X., Hong H., Cai T. (2017). Positron emission tomography (PET) guided glioblastoma targeting by a fullerene-based nanoplatform with fast renal clearance. Acta Biomater..

[192] Äärelä A., Auchynnikava T., Moisio O., Liljenbäck H., Andriana P., Iqbal I., Lehtimäki J., Rajander J., Salo H., Roivainen A. (2023). In Vivo Imaging of [60]Fullerene-Based Molecular Spherical Nucleic Acids by Positron Emission Tomography. Mol. Pharm..

[193] Bolskar R.D. (2008). Gadofullerene MRI contrast agents. Nanomedicine.

[194] Li T., Dorn H.C. (2017). Biomedical Applications of Metal-Encapsulated Fullerene Nanoparticles. Small.

[195] Kostyuk S.V., Proskurnina E.V., Ershova E.S., Kameneva L.V., Malinovskaya E.M., Savinova E.A., Sergeeva V.A., Umriukhin P.E., Dolgikh O.A., Khakina E.A. (2021). The Phosphonate Derivative of C_60_ Fullerene Induces Differentiation towards the Myogenic Lineage in Human Adipose-Derived Mesenchymal Stem Cells. Int. J. Mol. Sci..

[196] Kobzar O.L., Trush V.V., Tanchuk V.Y., Zhilenkov A.V., Troshin P.A., Vovk A.I. (2014). Fullerene derivatives as a new class of inhibitors of protein tyrosine phosphatases. Bioorg. Med. Chem. Lett..

